# A descriptive system for the Infant health-related Quality of life Instrument (IQI): Measuring health with a mobile app

**DOI:** 10.1371/journal.pone.0203276

**Published:** 2018-08-31

**Authors:** Ruslan Jabrayilov, Antoinette D. I. van Asselt, Karin M. Vermeulen, Sheri Volger, Patrick Detzel, Livia Dainelli, Paul F. M. Krabbe, for the Pediatrics expert group

**Affiliations:** 1 Department of Epidemiology, University of Groningen, University Medical Center Groningen, Groningen, The Netherlands; 2 Janssen Inc., Spring House, Pennsylvania, United States of America; 3 Nestlé Research Center, Nestlé, Lausanne, Switzerland; National University of Singapore, SINGAPORE

## Abstract

**Background:**

The assessment of health-related quality of life (HRQoL) is important for health outcomes research, disease modeling studies and comparisons of different healthcare interventions. Yet, only a few tools are available to assess HRQoL in 0-1-year-old infants. Furthermore, there is a need for an instrument able to assess HRQoL with a single, standardized, overall score in the first year of life. Here we described the development of the Infant health-related Quality of life Instrument (IQI), a generic, preference-based instrument that can be administered through a mobile application for assessing HRQoL in 0-1-year-old infants.

**Methods:**

A multi-step development process began by extracting candidate health concepts from relevant measures identified by two literature searches. Next, three panels, with experts from Asia, Europe, New Zealand and United States of America, and two surveys, with primary caregivers in New Zealand, Singapore, and the United Kingdom, evaluated the relevance of the candidate health concepts, organized them into attributes based on their similarities, explored alternative attributes and generated response scales. Additional interviews assessed the cross-cultural interpretability, parents’ understanding of health attributes, and the usability of the mobile application.

**Results:**

The final list of 7 health attributes included in the IQI consisted of *sleeping*, *feeding*, *breathing*, *stooling/poo*, *mood*, *skin*, and *interaction*. The users’ experiences with the mobile application were generally positive.

**Conclusions:**

The IQI is the first generic, preference-based, instrument designed to assess overall HRQoL in 0-1-year old infants. It is short and easy-to-administer through a mobile application. Moreover, close attention was paid to the opinions of the infants’ primary caregivers during the instrument and mobile application development process.

## Introduction

Over the past decades, advances in medical treatments have improved survival and reduced key morbidities, and treatment differences with regard to these traditional outcomes have diminished. As a consequence, health status or health-related quality of life (HRQoL) assessments are becoming more and more relevant. Regulatory bodies such as the Food and Drug Administration (FDA) [1] and National Institute for Health and Care Excellence (NICE) [2] actively encourage measuring patient-reported HRQoL in addition to traditional clinical assessments in healthcare.

HRQoL instruments can be developed based on different measurement frameworks. However, when comparing HRQoL across different populations, conducting disease modeling studies, and economic evaluations of various healthcare interventions, using a so-called preference-based instruments is more reasonable. Preference-based measures of HRQoL differ from other measures in that they, expressed in a single metric score, explicitly incorporate weights that reflect the importance attached to specific health aspects [3]. So far, no generic preference-based instrument exists for infants from 0 to 1 year of age. Even though one of the commonly used outcome measures in newborns, the neonatal Apgar score [4], expresses the child’s condition in one single score, it is more of a clinical measure than an HRQoL measure per se. In addition, the Apgar score is not preference-based. Other instruments such as the QUALIN [5], ITQOL [6, 7], PedsQL [8] and TAPQOL [9, 10] exist that can be used to measure HRQoL in infants but they are lengthy and may be tedious to complete for busy caregivers. More importantly, these instruments are composed of different sets of questions that yield distinct HRQoL scores for different health domains rather than a single, preference-based score. Currently, the instruments that are able to do this are only available for ages 4 and above [11, 12].

Most conventional methods to derive preference-based measures stem from health economics (e.g., standard gamble, time trade-off) and are susceptible to problems with their use due to flaws in adaptation, time preference, context, reference point, and other biases [13, 14, 15]. All economic methods use hypothetical health states that are assessed by a sample of (healthy) members of the general population. However, it is reasonable to assume that healthy people are not adequately informed or lack the imagination to appropriately judge the impact of health states, particularly severe ones, especially the health states of infants [16, 17]. Therefore, a new method to value health states was recently introduced. This measurement method, the multi-attribute preference response (MAPR) model, is based on the Rasch model (an item response theory model) [18, 19, 20]. The response mechanism of the MAPR model is less susceptible to various biases that conventional methods are prone to. Measurement with the MAPR model is based on a discrimination principle: a patient’s own health status, as classified in a first task, serves as a comparator state against other states in a second task. In case of small children, the parent serves as a proxy and provides this assessment. Because the response task in the MAPR model is simply a preference ranking between the infants’ own health status (that serves as a reference standard) and one or more (closely) related hypothetical health states, the assessment is rather easy to accomplish. However, for the seamless administration of this two-step procedure a pen and paper administered questionnaire is insufficient and instead, computerized assessment is essential.

Given the aforementioned limitations of available instruments, our aim was to develop a generic preference-based HRQoL instrument for infants between 0–1 years of age which includes health attributes relevant at each time point up to 1 year of age. In this paper we describe the multistep framework used to develop the Infant Quality of life Instrument (IQI) and its mobile application.

## Methods

### Literature search

The first critical task in the development of a preference-based HRQoL instrument for infants was to identify the HRQoL-related health concepts for this population. To this end, we conducted two literature searches. The first search identified articles containing both generic and disease-specific (for common childhood illnesses such as colic, regurgitations, asthma and eczema) HRQoL instruments, used in infants and children. The second search aimed to identify clinical scales and index instruments (e.g., checklists, questionnaires), mainly used in infant populations. The search terms can be found in S1 Table. It was assumed that these clinical measures covered concepts that are relevant to infant HRQoL. Both searches were restricted to articles in English written in the last 15 years, and to instruments for infants up to two years of age. Papers were selected first based on screening of the abstracts, and in a second stage based on the full text. Health attributes selected for extraction had to have observable characteristics and be applicable to each time point up to 1 year of age. Concepts could include, for example, lung function, crying and feeding problems. In case of uncertainty on the relevance, they were included in the list to be further evaluated in the expert rounds.

### Expert rounds

Meetings were held in the Netherlands and Greece, and via teleconference with the United States of America (USA) and New Zealand (NZ) with experts in various branches of pediatrics (see Acknowledegements for the list of experts). Each meeting, led by a moderator, included at least 3–4 experts and representatives of the research group. The meetings were held according to a fixed script, although procedures were adapted to fit each context (face to face or via teleconference). The objective of the project, as well as the expected role of the experts, was explained at the start of each meeting. Candidate health attributes were then discussed and evaluated for their relevance and importance to HRQoL in young infants.

During the expert meetings, the health concepts extracted from the two literature searches were grouped into health attributes (domains, aspects, dimensions, indicators etc.) and reviewed to assure they were age-appropriate and relevant to the clinicians. Two team members recorded the key points of the discussions. After the meeting, their notes were compared and attributes that were deemed unequivocally irrelevant for HRQoL of the infant population were excluded from the list and not discussed during later meetings. The notes also served as a basis for possible changes in the phrasing of certain health attributes or their levels. Based on this information health attributes were either retained, rephrased or eliminated.

### Surveys with primary caregivers

Two surveys were conducted among primary caregivers (called parents hereafter) from NZ, Singapore and the United Kingdom (UK). These countries were selected for practical reasons, since they are culturally different yet share one language, thus eliminating the need for translation at this phase and enabling the analysis of possible cross-cultural differences in the results. Parents were recruited by a marketing company (Survey Sampling International, SSI; www.surveysampling.com) from an online panel. All members of this panel have given their consent to participate in various studies. Only parents whose infant(s) were three years of age and younger were recruited. Parents aged below 18 or above 65 were excluded from the study. SSI contacted candidate respondents until the minimum sample size per country was reached. The Medical Ethics Review Committee at the University Medical Center of Groningen issued a waiver for this study, as the pertinent Dutch Legislation (the Medical Research Involving Human Subjects Act) does not apply to non-interventional studies (METc2017.115). Before the survey started, potential participants were informed about the purpose of the survey and the anonymity of their responses. By proceeding to the actual survey, informed consent was assumed. Both surveys were made in ViewletBuilder 8 Enterprise software (http://www.qbssoftware.com).

#### First survey

The purpose of the first survey was to obtain feedback from parents on the importance and relevance of the candidate infant health attributes proposed by the expert panels and to identify additional parent-generated attributes not previously considered. In order to explore possible new infant health attributes, the parents were first asked to record the three most relevant aspects of their infant’s health. The open-ended format of this question was intended to capture the parents’ perspective and their understanding of the concept of infant health. In the second task, the parents were asked to rank the candidate health attributes proposed by the experts from the most to the least important (S1 Slides).

#### Second survey

The main purpose of the second survey was to test the usability (e.g., clarity of instructions, user-friendliness and difficulty of the tasks) of the mobile application. Therefore, the parents first had to complete the two tasks in the mobile application (see next section) before answering the survey questions. Additionally, the parents were also given the opportunity to mention important infant health attributes not included in the mobile application in order to identify possible new ones (S2 Slides). Given the vast number of possible attributes that can be generated with an open-ended format, as well as the limitations in the number of attributes to be included in the final instrument, we only considered attributes mentioned by more than 10% of the respondents as relevant. Other questions in this survey asked parents to rate the relevance of previously identified attributes. Hence, the second survey was partly a replication of the first one.

Telephone interviews were conducted in a small sample of US, UK parents (10) to assess their understanding of the questions, the relevance of the IQI’s health attributes (concepts) and their experience completing the IQI on a computer or mobile device (instrument usability). The structured, qualitative, interviews were conducted approximately 48 hours after completing the IQI and followed a predefined protocol. During the interviews, questions related to the cross-cultural interpretability and parents’ understanding of individual health attributes (e.g., “What does the word …… mean to you?”; and “*Were there any words in the survey that you did not understand*?”), as well as the usability of the mobile application (e.g., “*Did the rotation of the boxes made Task 1 easier*?”) were asked. The approximate length of the interviews was 20 minutes.

### Mobile application

**Fig 1:**
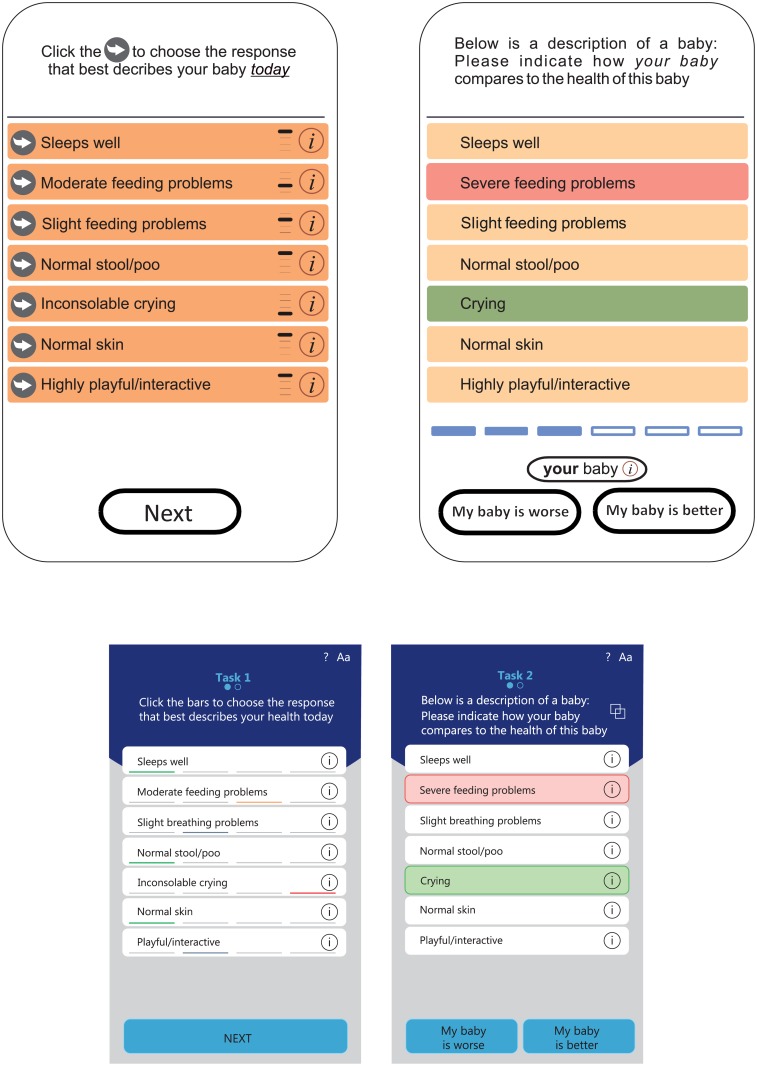
Screenshots of the prototype mobile application for the IQI. Left: task 1; right: task 2.

The mobile application consists of two tasks. In the first task, all health attributes are listed in interactive boxes, located in a table format on a single screen (Fig 1, left). By clicking on the interactive box for a specific attribute, the box rotates displaying the response options (see for an example: www.healthsnapp.info). For instance, when clicking on the box labeled ‘sleeping’, the box rotates and displays the response options ‘sleeps well’, ‘slightly affected sleep’, ‘moderately affected sleep’, and ‘severely disturbed sleep’. The users (i.e., parents) are asked to classify the health of their infant by rotating the boxes until the descriptions in all the boxes best describe their infants’ health status. The specific combination of responses (levels) chosen, constitutes the overall health state of their infant. In the second task, the infant health state, as defined in task 1, is compared to hypothetical infants with slightly different health states (Fig 1, right). Parents are then asked to choose whether the hypothetical child’s health is better or worse than the health of their own child. This procedure is the essence of a preference-based measurement, whereby individuals are asked to show their preferences for various health states [3, 21]. It is operated by a data collection technology (mobile application in combination with a central server) that is new in the field of HRQoL measurement, as it combines a newly developed measurement model with interactive software routines that are generic and flexible [20]. Currently, the English version of the mobile application of the IQI is available (www.healthsnapp.info).

## Results

### Literature search

The first literature search identified 235 unique health attributes (S1 Fig). Out of these, 79 were excluded: 17 for not being applicable to 0-1-year-old infants (e.g., ‘missing out on normal childhood activities’, or ‘feeling different from others’), 2 for being too disease-specific (e.g., ‘immunization’ and ‘thick leathery skin’), 43 for not reflecting the perspective of the infant but rather the effect of his/her condition on the parents (e.g., the ‘effect of baby’s condition on family members’), 12 for reflecting the result of the disease rather than the disease itself, 3 for being largely overlapping and 1 for being too broad. One attribute appeared in 2 categories simultaneously and hence was deleted from one of them.

The second literature search identified 138 attributes, extracted from clinical scales and index instruments. In total, 59 of those were excluded; 19 for being already included in the first search and 40 for partly the same reasons as in first literature search, and partly because of relevance of the attributes to the context of a disease (e.g., parents’ knowledge of the child’s disease, professional help involved) rather than to the disease itself.

The final set consisted of 235 candidate infant health attributes: 156 from the first literature search and 79 from the second.

### Expert rounds

The average duration of the expert meetings was approximately two hours, with no difference in duration between meetings and telephone conferences. The first meeting (Netherlands) was attended by two pediatricians and one pediatric speech therapist, the second (Greece) by 4 pediatricians, and the last one (telephone conference) by three pediatricians. The experts, from Asia, Europe, NZ and USA, were specialized in various branches of medicine, i.e., gastroenterology, endocrinology, nutrition, neonatal neurology, pulmonology, nursing sciences and speech therapy. Each session was guided by at least 3 team members and chaired by one of the primary investigators.

During expert meetings the following 8 health attributes were identified as potentially relevant: *sleeping*, *feeding*, *breathing*, *stooling*, *mood*, *skin*, *spitting*, and *general discomfort*. Although some experts regarded *spitting* as irrelevant, others were unsure whether it should be removed from the list. Therefore, it was retained until more data were available from parent surveys.

### Parent surveys

#### First survey

A total of 1392 parents from NZ (26%), Singapore (31%) and the UK (42%) were recruited (S1 Data). The mean (SD) age of the parents was 31.2 (6.1) years [NZ 30.5 (6.4); Singapore 31.4 (5.5); UK 31.6 (6.8)]. Infants’ ages ranged between 0–1 years (38%), 1–2 years (32%) and 2–3 years (30%).

*Sleeping*, *feeding*, *health* and *playing* were the attributes most frequently mentioned by parents in task 1 (Table 1). Of these, *sleeping* and *feeding* were also considered highly relevant by the expert panels. The other attributes, *health* and *playing*, were mentioned by 15% and 14% of the parents, respectively. However, *health* was not considered as an attribute in itself because the term *health* was considered too broad. Attributes mentioned by less than 10% of the parents did not form specific categories. Rather, they fell into general categories such as of *food/nutrition/diet*, *socializing/interaction*, *being active/exercising*, *happiness/love*, *learning/brain development* (see Additional file 3 for computational details for the grouping of attributes into categories).

**Table 1:** Health attributes mentioned by more than 10% of parents in an open-ended format (survey 1).

		New Zealand n = 368 (%)	Singapore n = 425 (%)	United Kingdom n = 598 (%)	Total sample n = 1391 (%)
1.	Sleeping	291 (79)	301 (71)	455 (76)	1047 (75)
2.	Feeding	214 (58)	221 (52)	341 (57)	776 (56)
3.	Health	48 (13)	65 (15)	94 (16)	207 (15)
4.	Playing	41 (11)	43 (10)	122 (20)	206 (15)

In the ranking task (Table 2), *sleeping*, *feeding* and *breathing* were ranked as the top 3 most important health attributes irrespective of country or age of the infants (S1 Appendix). Similarly, *spitting* was ranked as the least important in all the countries and infant age groups. These findings confirmed the experts’ doubts regarding the relevance and importance of *spitting* as an infant health attribute.

**Table 2:** Mean rankings of the health attributes by importance in three different countries (Survey 1).

New Zealand (n = 368)		Singapore (n = 425)		United Kingdom (n = 598)		Total sample (n = 1391)	
Attribute	Mean rank ^a^^,^ ^b^	Attribute	Mean rank ^a^^,^ ^b^	Attribute	Mean rank ^a^^,^ ^b^	Attribute	Mean rank ^a^^,^ ^b^
Breathing	1.83	Sleeping	2.42	Breathing	2.03	Breathing	2.03
Feeding	2.51	Breathing	3.49	Feeding	2.74	Sleeping	2.74
Sleeping	2.90	Feeding	3.55	Sleeping	3.05	Feeding	3.05
Mood	4.95	Stooling/poo	4.23	Mood	4.59	Mood	4.59
General discomfort	5.07	General discomfort	4.76	Stooling/poo	5.16	Stooling/poo	5.16
Stooling/poo	5.26	Mood	4.78	General discomfort	5.18	General discomfort	5.18
Skin	5.60	Skin	5.44	Skin	5.52	Skin	5.52
Spitting	7.51	Spitting	7.02	Spitting	7.52	Spitting	7.52

^a^ The rankings range from 1 to 8.

^b^ Higher rankings indicate less importance.

#### Second survey

A total of 158 parents from NZ (31%), Singapore (36%) and the UK (33%) were contacted (S6 Data). Infants’ ages ranged between 0–1 years (55%), 1–2 years (41%) and 2–3 years (4%). On a scale from 1 (Completely Disagree/Never) to 5 (Completely Agree/Always), the mean ratings for the usability and understandability statements of the mobile application were generally above 3, indicating a choice between *neutral* and *agree* (Table 3). Based on these results and the suggestions given during the follow-up phone interviews, the usability and understandability of the mobile application were further improved.

**Table 3:** Usability assessment of the mobile application*.

	Statement ^a^	Country			
		New Zealand	Singapore	United Kingdom	Total
Task 1	1. Instructions were understandable	3.48(.22)	3.83(.26)	3.68(.24)	3.70(.24)
	2. Rotation of the boxes made sense to me	3.88(.30)	3.65(.28)	3.35(.26)	3.66(.28)
	3. Task 1 required much effort	3.37(.20)	3.74(.18)	3.46(.22)	3.49(.18)
	4. I made use of the help screens ^b^	2.72(.32)	2.47(.33)	2.90(0.30)	2.73(.32)
Task 2	1. Instructions were understandable	3.58(.22)	4.07(.24)	3.65(.26)	3.81(.24)
	2. I was able to guess why boxes were red and green	3.71(.15)	4.18(.13)	3.41(.17)	3.80(.15)
	3. The “your infant” screen was useful	3.82(.24)	4.11(.26)	3.54(.22)	3.85(.24)

*^Mean (SD)^

^a^ 1—Strongly Disagree, 2—Disagree, 3—Neutral, 4—Agree, 5—Completely Agree

^b^ 1—Never, 2-Rarely, 3—Sometimes, 4—Often, 5 –Always

Parents were also asked to rank the revised health attributes, included *playing* which was identified as relevant to parents in Survey 1. In this survey *spitting*, *stooling*, *general discomfort* and *playing* were consistently rated by parents as the least relevant infant health attributes. Interviews with 10 parents confirmed that these attributes were not entirely clear and feedback was given to either rename them or to better define them. Based on this input and considering the results of the first survey, as well as previous expert recommendations, *spitting* and *general discomfort* were removed from the list. Furthermore, *stooling* and *playing* were reworded to *stooling/poo* and *interaction*, respectively, to improve their cross-cultural interpretability and relevance across English speaking countries. Finally, the term *interaction* was considered more appropriate than playing for infants aged 0–12 months.

**Fig 2:**
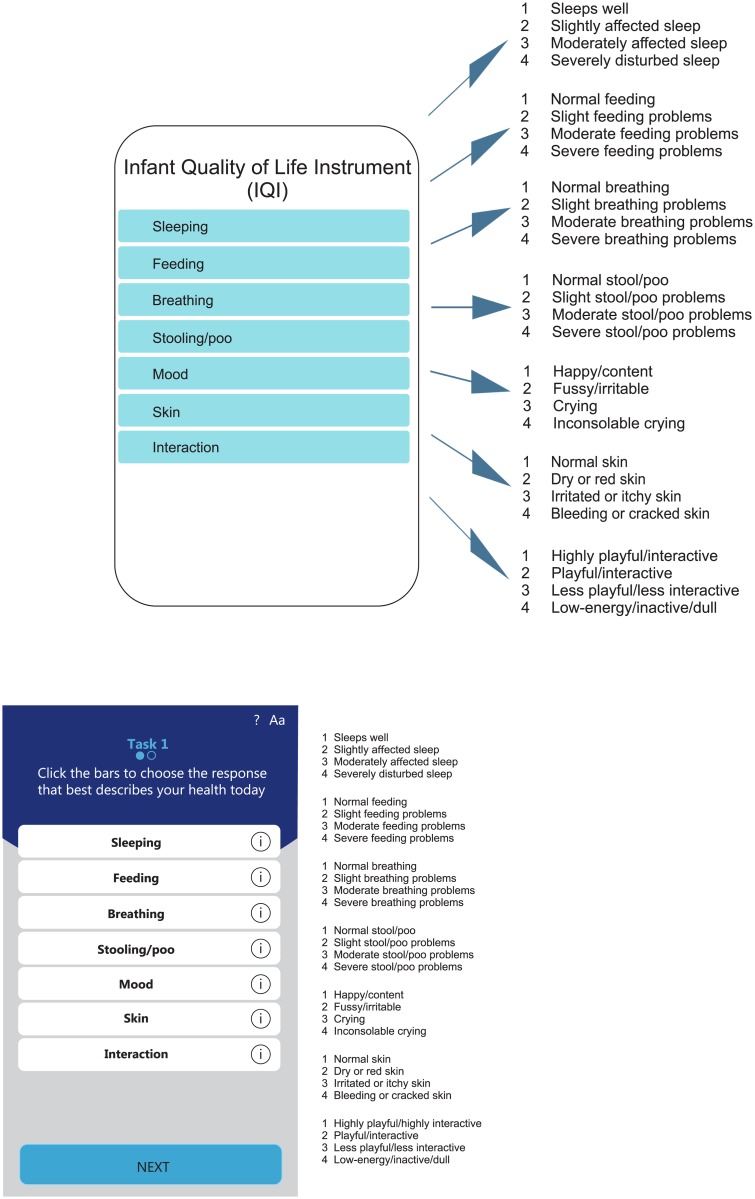
Final health attributes and their levels included in the IQI.

Fig 2 shows the final list of health attributes included in IQI: *sleeping*, *feeding*, *breathing*, *stooling/poo*, *mood*, *skin*, *interaction*. Answers from parents of children older than the age range of interest (0–1 year) were in line with the others, which means that the selected attributes are consistent over time; even though they were judging their infants retrospectively, they still considered the attributes important, making the selection of the attributes even more reliable.

## Discussion

We developed the first generic, preference-based instrument to assess HRQol of infants 0-1-year-old with a mobile application. This new instrument overcomes many of the limitations of the HRQoL instruments [5–10] currently in use.

First, the IQI is embedded in a preference-based measurement framework that enables capturing HRQoL in a single score. The two tasks that the parents had to complete in the mobile application in the second survey, i.e. rating the health of their infants and comparing it to those of other infants, forms the basis of preference-based measurement [3, 21] (It should be noted, however, that the data gathered from these tasks were used solely for testing the usability of the mobile application). IQI scores are expressed on a relative scale and can be used for many clinical purposes. In addition, supplementary studies are planned whereby the scores will be transformed to an anchor-based range (from 0 = death to 1 = full health). After this transformation, it will also be possible to compute quality-adjusted life years (QALYs) that are necessary for economic evaluations of different healthcare programs for infants.

Second, the IQI was developed based on comprehensive literature reviews, expert opinion and extensive input from parents. This ensures that it captures the most relevant health attributes in 0-1-year-old infants from various perspectives. One of the limitations of the currently available infant HRQoL instruments is that the age-range they cover may be too wide for certain attributes, thus inappropriate for infant populations in a specific age group. For example, the ITQOL [6, 7] contains physical ability items such as *grasping* or *reaching* that are likely not applicable for the older infants (> 9 months) while items such as *walking* and *running* are rarely relevant for younger ones (< 9 months). Taking into account the opinions of the parents proved to be useful in spotting health attributes that were irrelevant (e.g., *general discomfort*) and discovering new ones (e.g., *interaction*) relevant to 0-1-year-old infants.

Traditional generic HRQoL instruments—based on classical test theory—consist of one or more health domains, each measured by multiple items [3, 22]. The measurement properties of these types of instruments are typically established using various psychometric reliability (e.g., internal consistency, test-retest) and validity (e.g., factor analysis, convergent/discriminant/criterion validity) analyses. While, in preference-based measurement, the task is focused on evaluating preferences to (hypothetical) health states based on a small number of distinct health attributes to generate another type of health outcome, namely health-state values. As a consequence, many of the psychometric statistics used to evaluate reliability and validity properties of traditional instruments, are not well-suited for the development and evaluation of preference-based instruments [23]. The many challenges associated with establishing the content validity of PROs, especially preference-based measures, are recognized by the field of health instrument development and highlighted in the Food and Drug Administration’s PROM industry guidance [1]. The solution might lie in establishing special procedures to retrieve the relevant attributes, such as by consulting expert opinion, special judgment procedures [24], or re-examining the content of existing instruments. Because the aim of the study was to establish the conceptual framework of the IQI, select relevant health attributes and to conduct usability testing, extensive validation of the IQI instrument was considered out of the scope for this study. Future studies are planned to evaluate the IQI’s psychometric measurement properties.

A purposeful feature of IQI is that it is short and easy-to-administer. Current HRQoL instruments for infants tend to be lengthy, paper and pencil-based and can place a high burden on busy parents. Recently, computer adaptive testing has successfully been used to measure HRQoL instruments and reduce respondent burden [25]. Similarly, the IQI’s mobile application provides an opportunity to streamline the instrument’s development process. Given the interactive nature of the IQI’s tasks, administration with a mobile application—as opposed to paper and pencil testing- is almost a necessity. For example, in task 2, parents are presented hypothetical infant health states closely related to actual infants’ health states obtained in task 1. In other words, the output of task 1 is used as input for task 2 and therefore, a seamless transition between the two tasks is only possible through an automated interactive process.

Typically, preference-based instruments are based on a single, small but complicated study designed to derive the utility weights for the instrument’s attributes. Subsequently, the descriptive content of these instruments can be administered simply by paper and pencil. For the IQI’s measurement framework the descriptive content and the preference tasks are united. Therefore, the precision of the weights will increase with additional responses and ultimately even the interactions between attributes can be estimated. A mobile application permits rapid, centralized storage of item responses, the addition of interactive explanation and instructional elements and feedback modules, and individual (utility) weights are automatically computed. In addition, a mobile application format allows for the extension of the MAPR model and its application, in which respondents may select some attributes from a larger set of candidate attributes [20].

That fact that IQI can be scored by parents is in line with the concept of patient reported outcome measurement (PROM). Use of PROM has the potential to improve the quality of healthcare because it facilitates a dialogue between doctors and patients (i.e., parents) which leads to more informed treatment decisions. In addition, taking into account the views of patients eliminate observer bias [26]. However, a limitation of our study might be the reliance on parents’ (proxy) opinions for measuring HRQoL in their infants. Individual differences between parents can result in different ratings of their child’s HRQoL. Other variables, such as gender, cultural attitudes, and values and perceptions of parents’ own health and quality of life in general, may also affect the ratings. Therefore, measurement equivalence across various populations has to be established for an effective and accurate measurement of HRQoL in infants. This does not apply only to IQI, but is a rather general requirement of all HRQoL instruments. Future studies should investigate whether the HRQoL values obtained with IQI are comparable across, for example, gender, age, and cultural background of the parents.

## Conclusions

The IQI is the first generic, preference-based HRQoL instrument designed to assess overall HRQoL in 0-1-year-old infants through an efficient mobile application. The IQI was developed with novel measurement methodology and the collective efforts of academic and industry researchers, international pediatrics experts and caregivers. The IQI mobile application was shown to be relevant, easy-to-use and well-understood in a sample of parents of infants 0–1 years old across Singapore, UK and USA. The results of this study support the further development of the instrument’s psychometric properties and various studies to arrive at weights for the levels of the 7 attributes of the IQI are planned.

## Supporting information

S1 Appendix Computational details for the open question and the ranking task. (DOCX)

S1 Data First survey. (CSV)

S2 Data Second survey. (CSV)

S1 Fig Flowchart of the first and the second literature studies. Left: study 1; right: study 2.
(DOCX)

S1 Slides Screens of the first survey. (PDF)

S2 Slides Screens of the second survey. (PDF)

S1 Table Search terms used in literature studies 1 and 2. Top: HRQoL instuments (study 1); bottom: clinical scales and index scores (study 2).
(DOCX)

## References

[1] Food and Drug Administration. Guidance for Industry Use in Medical Product Development to Support Labeling Claims Guidance for Industry, Guidance for Industry Patient-Reported Outcome Measures: Use in Medical Product Development to Support Labeling Claims. Silver Spring, MD; 2009. 10.1186/1477-7525-4-79 PMC1629006 17034633

[2] NICE National Institute for Health and Care Excellence. Guide to the methods of technology appraisal 2013 https://www.nice.org.uk/process/pmg9/chapter/foreword. Accessed 8 July 2018. 27905712

[3] Krabbe PFM. The measurement of health and health status: Concepts, methods and applications from a multidisciplinary perspective. 1st ed San Diego, USA: Elsevier/Academic Press; 2016.

[4] Apgar VA. Proposal for a new method of evaluation of the newborn Infant. Anesth Analg. 1953; 32: 260–267. 10.1213/ANE.0b013e31829bdc5c 13083014

[5] Manificat S, Dazord A, Langue J, Danjou G, Bauche P, Bovet F, et al Evaluation de la qualité de vie du nourrisson et du très jeune enfant: Validation d’un questionnaire. Étude multicentrique européenne [Evaluation of the quality of life of infants and very young children: validation of a questionnaire. Multicenter European study]. Arch Pediatr. 2000; 7: 605‐14. 10911526 10.1016/s0929-693x(00)80127-x

[6] Landgraf JM, Vogel I, Oostenbrink R, van Baar BM, Raat H. Parent-reported health outcomes in infants/toddlers: measurement properties and clinical validity of the ITQOL-SF47. Qual Life Res. 2013; 22: 635–646. 10.1007/s11136-012-0177-8 22528242

[7] Raat H, Landgraf JM, Oostenbrink R, Moll HA, Essink-Bot ML. Reliability and validity of the Infant and Toddler Quality of Life Questionnaire (ITQOL) in a general population and respiratory disease sample. Qual Life Res. 2016; 16: 445–460. 10.1007/s11136-006-9134-8 17111231 PMC2792359

[8] Varni JW, Limbers CA, Neighbors K, Schulz K, Lieu JE, Heffer RW et al The PedsQL Infant Scales: feasibility, internal consistency, reliability, and validity in healthy and ill infants. Qual Life Res. 2011; 20: 45–55. 10.1007/s11136-010-9730-5 20730626

[9] Fekkes M, Theunissen NC, Brugman E, Veen S, Verrips EG, Koopman HM, et al Development and psychometric evaluation of the TAPQOL: a health‐related quality of life instrument for 1‐5‐year‐old children. Qual Life Res. 2000;9: 961‐972. 11284215 10.1023/a:1008981603178

[10] Bunge EM, Essink-Bot ML, Kobussen MP, van Suijlekom-Smit LW, Moll HA, Raat H. Reliability and validity of health status measurement by the TAPQOL. Archiv Dis Child. 2005;90: 351‐358. 10.1136/adc.2003.048645 15781921 PMC1720358

[11] Stevens K. Valuation of the Child Health Utility 9D Index. Pharmacoeconomics. 2012; 30: 729–47. 10.2165/11599120-000000000-00000 22788262

[12] Chen G, Ratcliffe J. A review of the development and application of generic multi-attribute utility instruments for pediatric populations. Pharmacoeconomics. 2015;33: 1013–1028. 10.1007/s40273-015-0286-7 25985933

[13] Nord E, Enge AU, Gundersen V: QALYs: is the value of treatment proportional to the size of the health gain? Health Econ 2010; 19: 596–607. 10.1002/hec.1497 19459186

[14] Attema AE, Edelaar-Peeters Y, Versteegh MM, Stolk EA. Time trade-off: One methodology, different methods. Eur J Health Econ. 2013, 14(Suppl 1): 53–64. 10.1007/s10198-013-0508-x 23900665 PMC3728453

[15] Salomon JA: Techniques for valuing health states In Culyer AJ editor. Encyclopedia of Health Economics (vol. 2). San Diego: Elsevier; 2014 pp 454–458.

[16] Brazier JE, Dixon S, Ratcliffe J: The role of patient preferences in cost-effectiveness analysis: a conflict of values? Pharmacoeconomics 2009, 27:705–712. 10.2165/11314840-000000000-00000 19757864

[17] Krabbe PFM, Tromp N, Ruers TJM, van Riel PLCM: Are patients’ judgments of health status really different from the general population? Health Qual Life Outcomes 2011, 9(31). 10.1186/1477-7525-9-31 21569351 PMC3113921

[18] Krabbe PFM. A generalized measurement model to quantify health: The Multi-Attribute Preference Response Model. PLoS ONE. 2013;8(11):e79494 10.1371/journal.pone.0079494 24278141 PMC3836915

[19] Krabbe PFM. A generalized measurement model to quantify health: the multi-attribute preference response model In: Badiru AB, Racz LA, editors. Handbook of measurements: Benchmarks for systems accuracy and precision. Boca Raton: CRC Press; 2015.

[20] Groothuis-Oudshoorn CGM, van der Heuvel E, Krabbe PFM. A preference-based item response theory model to measure health: concept and mathematics of the multi-attribute preference response model. BMC Med Res Methodol. 2018; 18: 62 10.1186/s12874-018-0516-8 29929469 PMC6013962

[21] Brazier J, Ratcliffe J, Salomon J, Tsuchiya A. Measuring and valuing health benefits for economic evaluation. Oxford, UK: Oxford University Press; 2017.

[22] Varni JW, Seid M, Kurtin PS. PedsQL 4.0: Reliability and validity of the Pediatric Quality of Life Inventory Version 4.0 Generic Core Scales in healthy and patient populations. Med Care 2001; 39: 800–812. 10.1097/00005650-200108000-00006 11468499

[23] Stevens K, Palfreyman S. The use of qualitative methods in developing the descriptive systems of preference-based measures of health-related quality of life for use in economic evaluation. Value Health. 2012; 15: 991–998. 10.1016/j.jval.2012.08.2204 23244799

[24] Reneman MF, Brandsema KPD, Schrier E, Dijkstra PU, Krabbe PFM. Patients first: Towards a patient-centered, instrument to measure impact of chronic pain. Phys Ther. 2018; 98: 616–625. 10.1093/ptj/pzy040 29939365 PMC6181955

[25] Papuga MO, Mesfin A, Molinari R, Rubery PT. Correlation of PROMIS Physical Function and Pain CAT instruments with Oswestry Disability Index and Neck Disability Index in spine patients. Spine (Phila Pa 1976) 2017; 41: 1153–1159. 10.1097/BRS.0000000000001518 26909832 PMC4938742

[26] Black N. Patient reported outcome measures could help transform healthcare. BMJ. 2013; 346:f167 10.1136/bmj.f167 23358487

