# Supplementary material for: A descriptive system for the Infant health-related Quality of life Instrument (IQI): Measuring health with a mobile app
Source: PLoS One. 2018 Aug 31;13(8):e0203276. doi: 10.1371/journal.pone.0203276 (PMC6118381; doi:10.1371/journal.pone.0203276)
Supplement: S1 Appendix — (DOCX) [file pone.0203276.s001.docx]

In order to explore clusters of similar health attributes proposed by parents, an algorithm containing several steps was developed in the R programming language. First, the words were grouped together based on their 1st letter. This resulted in clustering of words such as Sleep and Sleeping but also words such as Stooling. Therefore, the procedure was repeated until a meaningful grouping of words was achieved. More specifically, the words were grouped based on the subsequent letters they contained. This ensured the separation of clusters containing Sleeping and Stooling because of the difference in their 2nd letter (i.e., Sl versus St). Moreover, the algorithm ensured grouping of the same words written in different letter cases (i.e., Sleeping, SLEEPING, sleeping and sLEEPING). Finally, different clusters expressing similar health attributes (i.e., stooling and excrement) were grouped together based on visual inspection.

The average rankings were computed in the following way. For each health attribute, the number of parents was first multiplied by the rank number (𝑁𝑟 × 𝑟,=1,2,3…8). Then the products were summed up and divided by the total 𝑛 to compute average rankings. For example, if 100 parents ranked Sleeping as number 1 (most important) and 90 parents as number 2, one would first add the products (100 × 1+ 90 ×2=280) and divide the result by the number of parents (100+90=190 in this example). The average ranking for Sleeping would then be 280/190=1.47.
