# Supplementary figures and images for: A descriptive system for the Infant health-related Quality of life Instrument (IQI): Measuring health with a mobile app
Source: PLoS One. 2018 Aug 31;13(8):e0203276. doi: 10.1371/journal.pone.0203276 (PMC6118381; doi:10.1371/journal.pone.0203276)

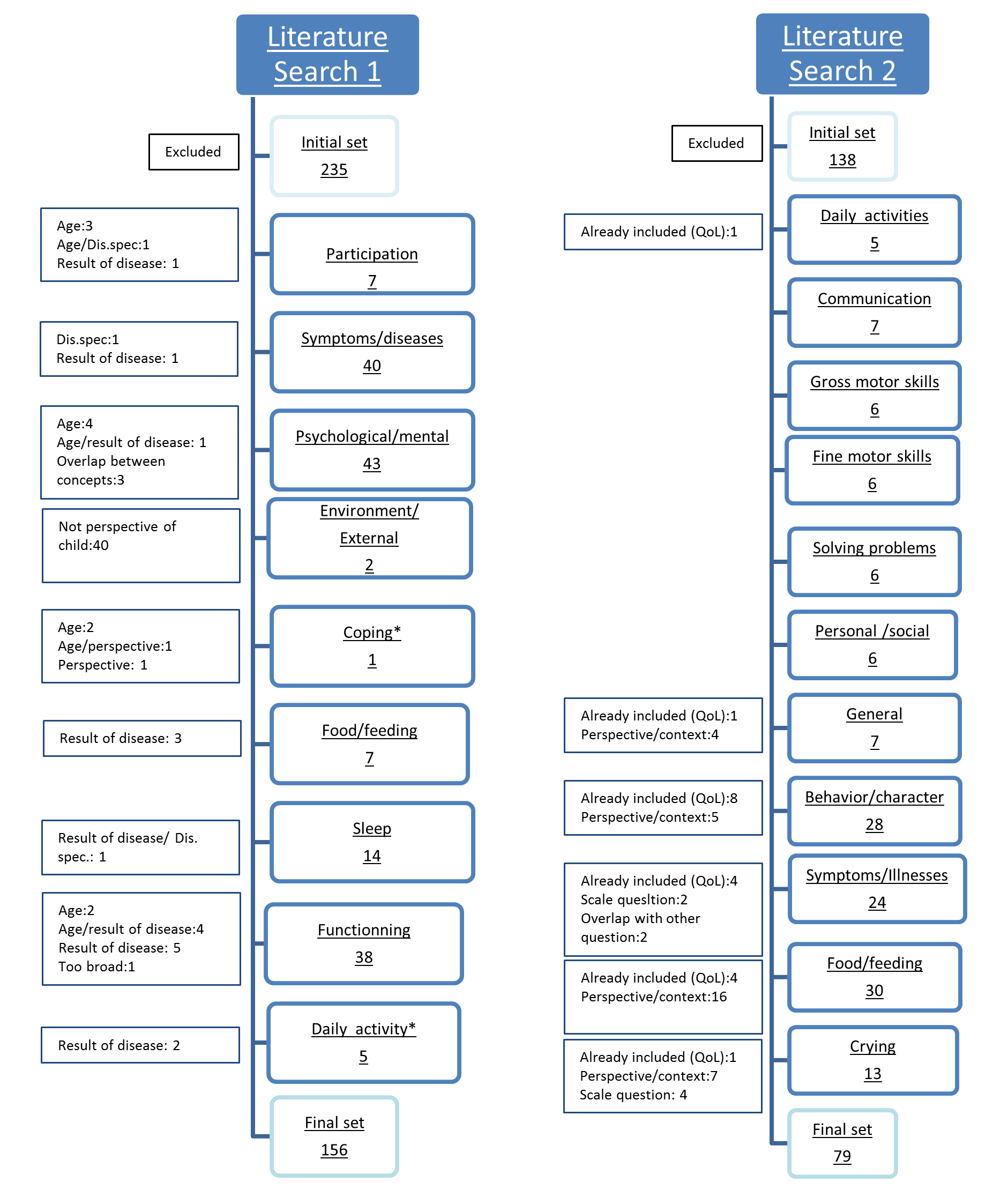


* Attribute appeared in *Coping* as well as in *Daily activity*.

Supplement: S1 Fig — Left: study 1; right: study 2. (DOCX) [file pone.0203276.s004.docx]
