# Supplementary material for: A descriptive system for the Infant health-related Quality of life Instrument (IQI): Measuring health with a mobile app
Source: PLoS One. 2018 Aug 31;13(8):e0203276. doi: 10.1371/journal.pone.0203276 (PMC6118381; doi:10.1371/journal.pone.0203276)
Supplement: S1 Slides — (PDF) [file pone.0203276.s005.pdf]

Introduction

This survey is intended to identify those aspects of your child's health and wellbeing that you consider most important. Your views will be incorporated in a questionnaire on quality of life in infants, which is under development by the University Medical Center Groningen, The Netherlands, in collaboration with Nestlé.

Please read carefully before you answer the questions. The survey will take approximately 10 minutes. We appreciate your time and effort in taking this survey!

Start Survey

umcg Nestlé

Please write down 3 aspects of well-being in your infant that you consider important and relevant. For example, crying, feeding etc.

1. ______________________

2. ______________________

3. ______________________

Submit

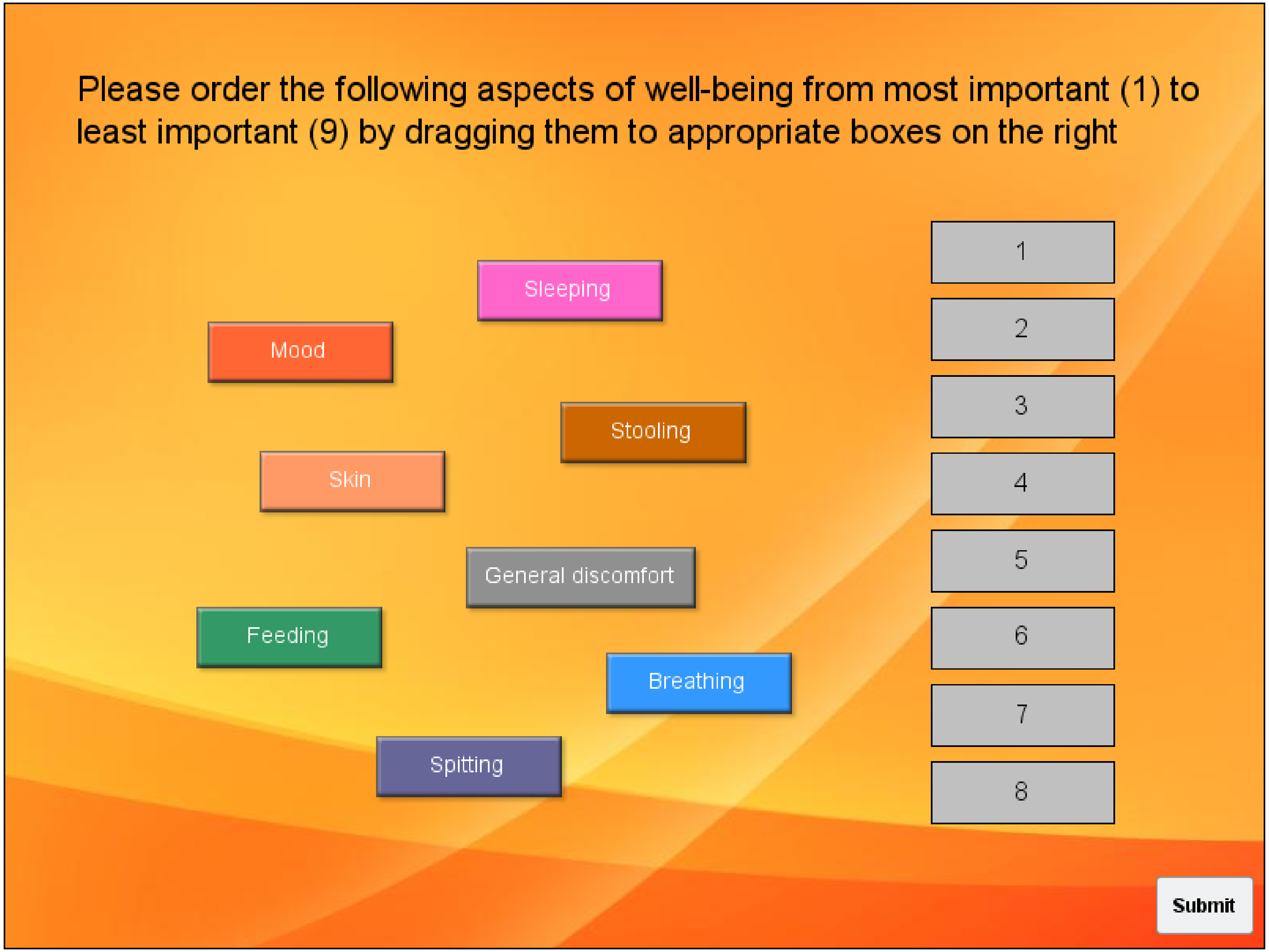
Please order the following aspects of well-being from most important (1) to least important (9) by dragging them to appropriate boxes on the right
Sleeping
Mood
Stooling
Skin
General discomfort
Feeding
Breathing
Spitting
1
2
3
4
5
6
7
8
Submit

Are there other aspects of well-being in your infant that you consider important or relevant?

1. ____________________
2. ____________________
3. ____________________

Submit

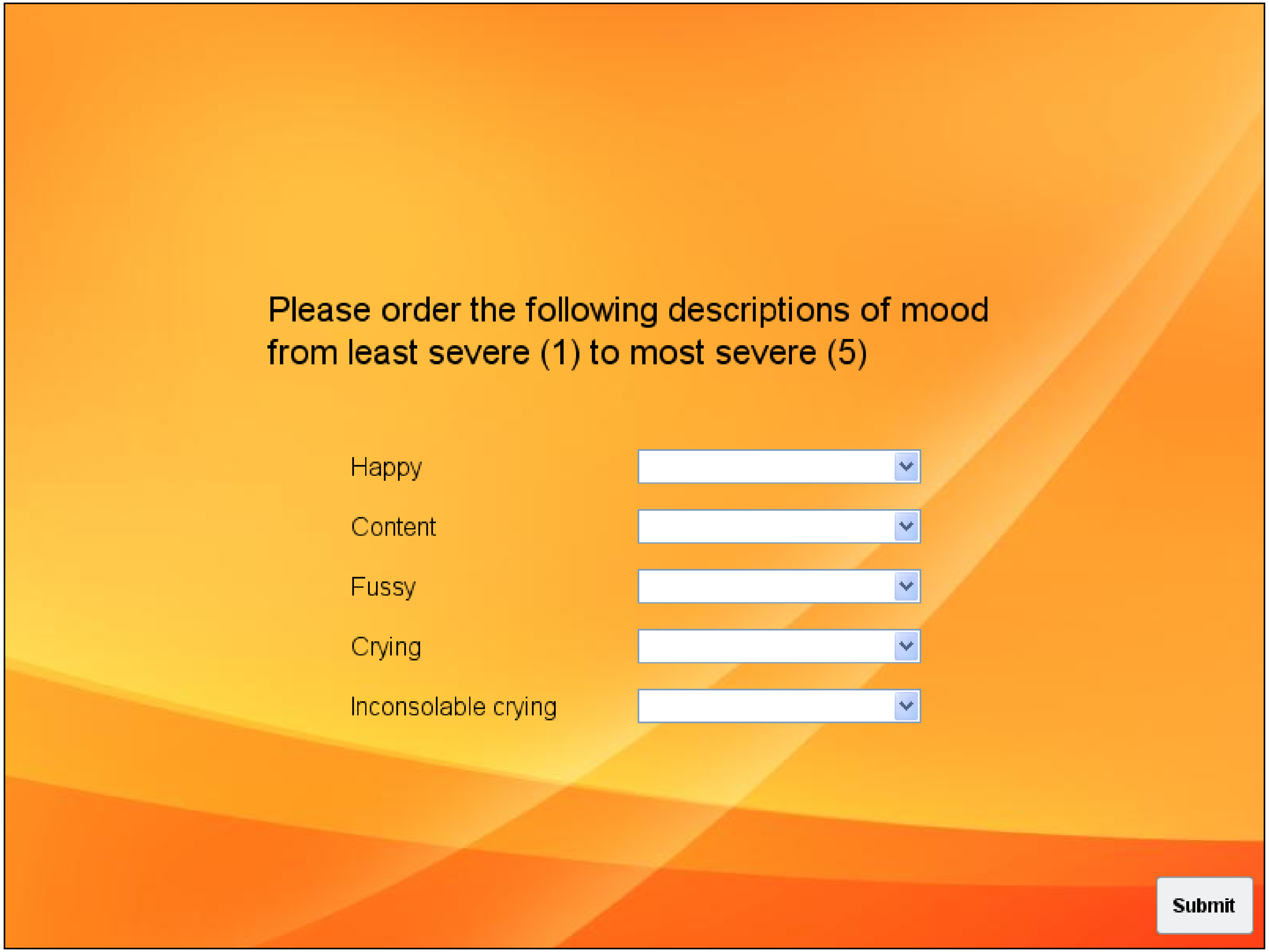
Please order the following descriptions of mood from least severe (1) to most severe (5)
Happy
Content
Fussy
Crying
Inconsolable crying
Submit

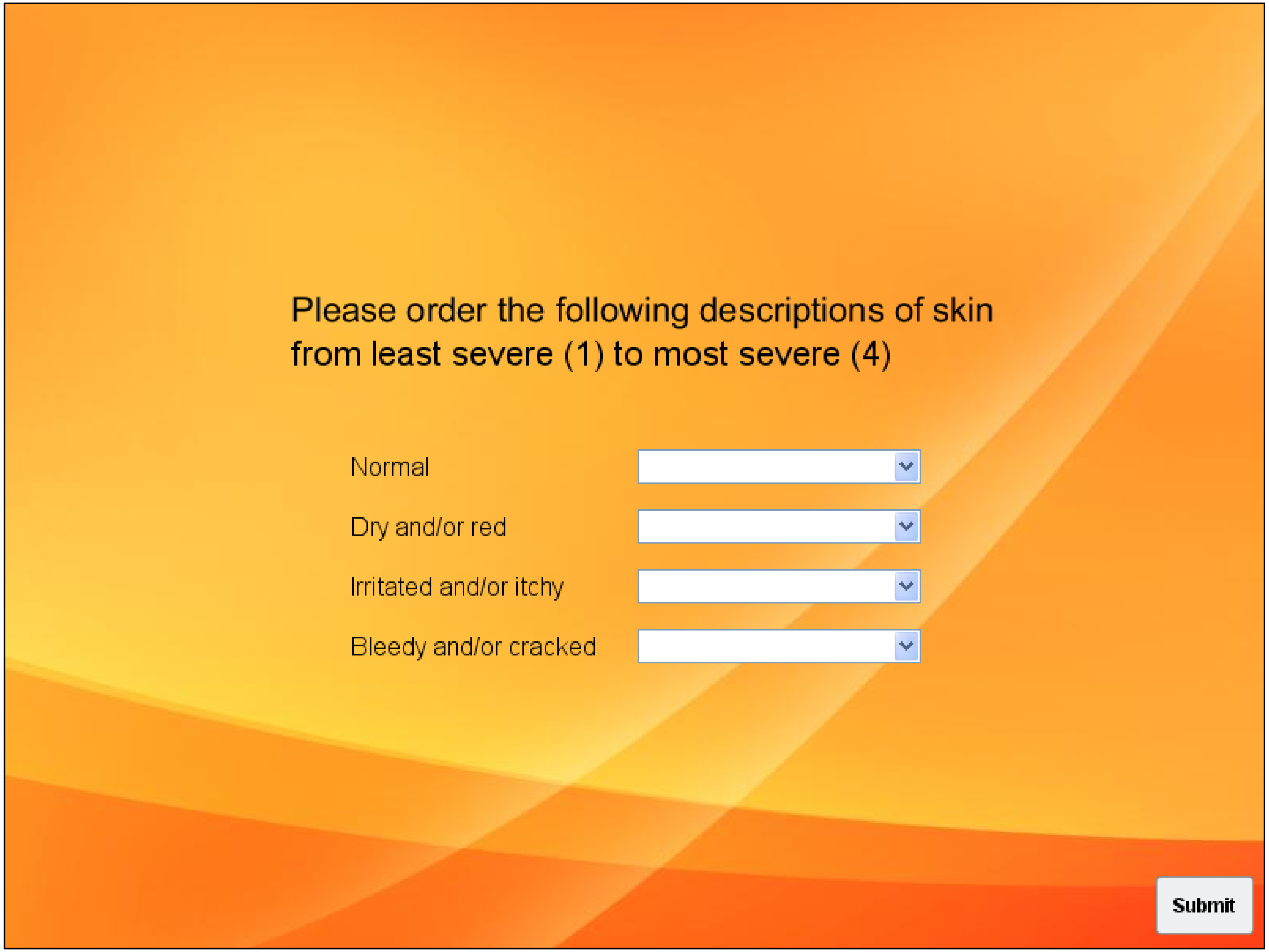
Please order the following descriptions of skin from least severe (1) to most severe (4)
Normal
Dry and/or red
Irritated and/or itchy
Bleedy and/or cracked
Submit

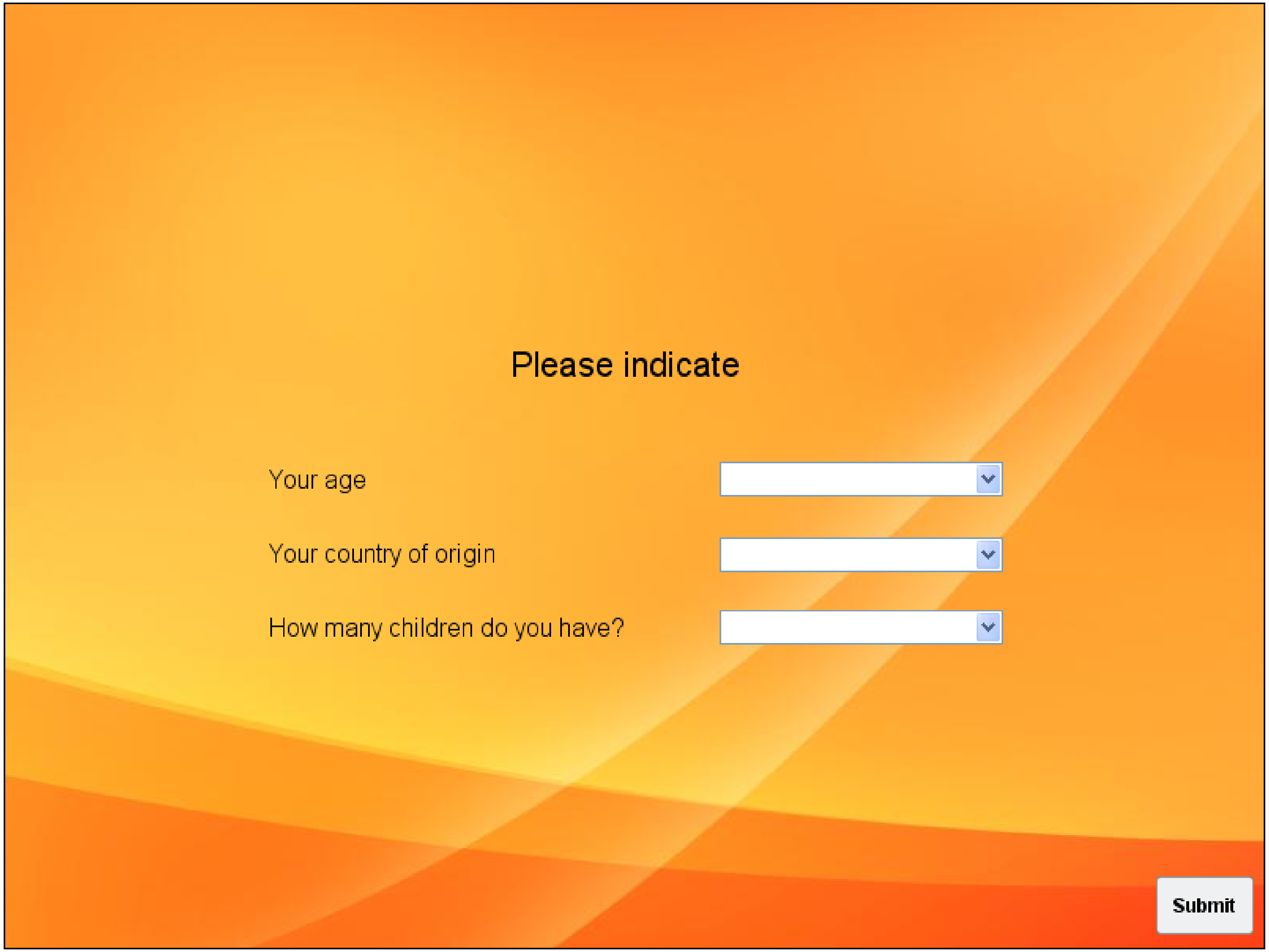

Please indicate

Your age

Your country of origin

How many children do you have?

Submit

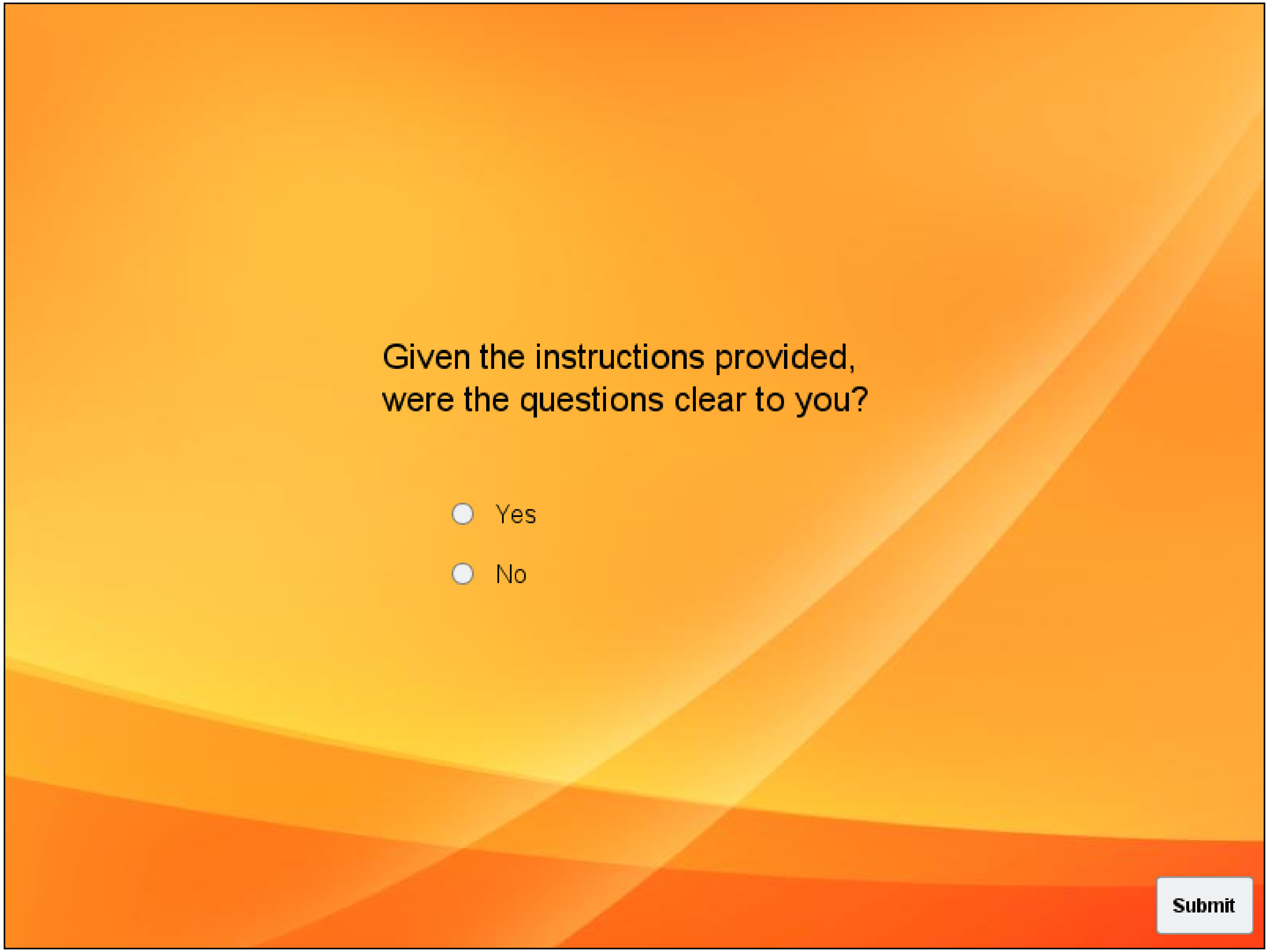
Given the instructions provided,
were the questions clear to you?
Yes
No
Submit

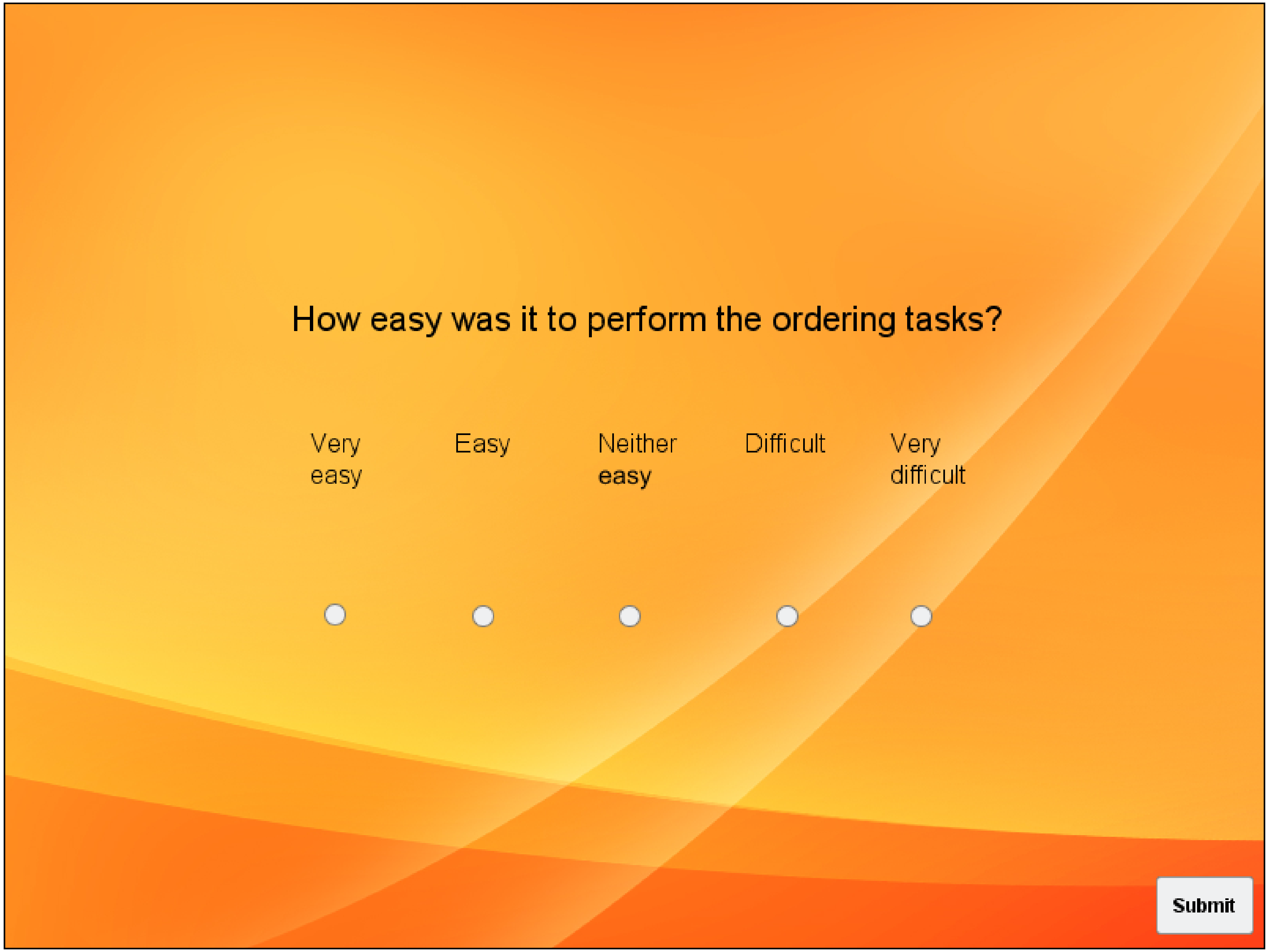
How easy was it to perform the ordering tasks?
Very easy
Easy
Neither easy
Difficult
Very difficult
Submit

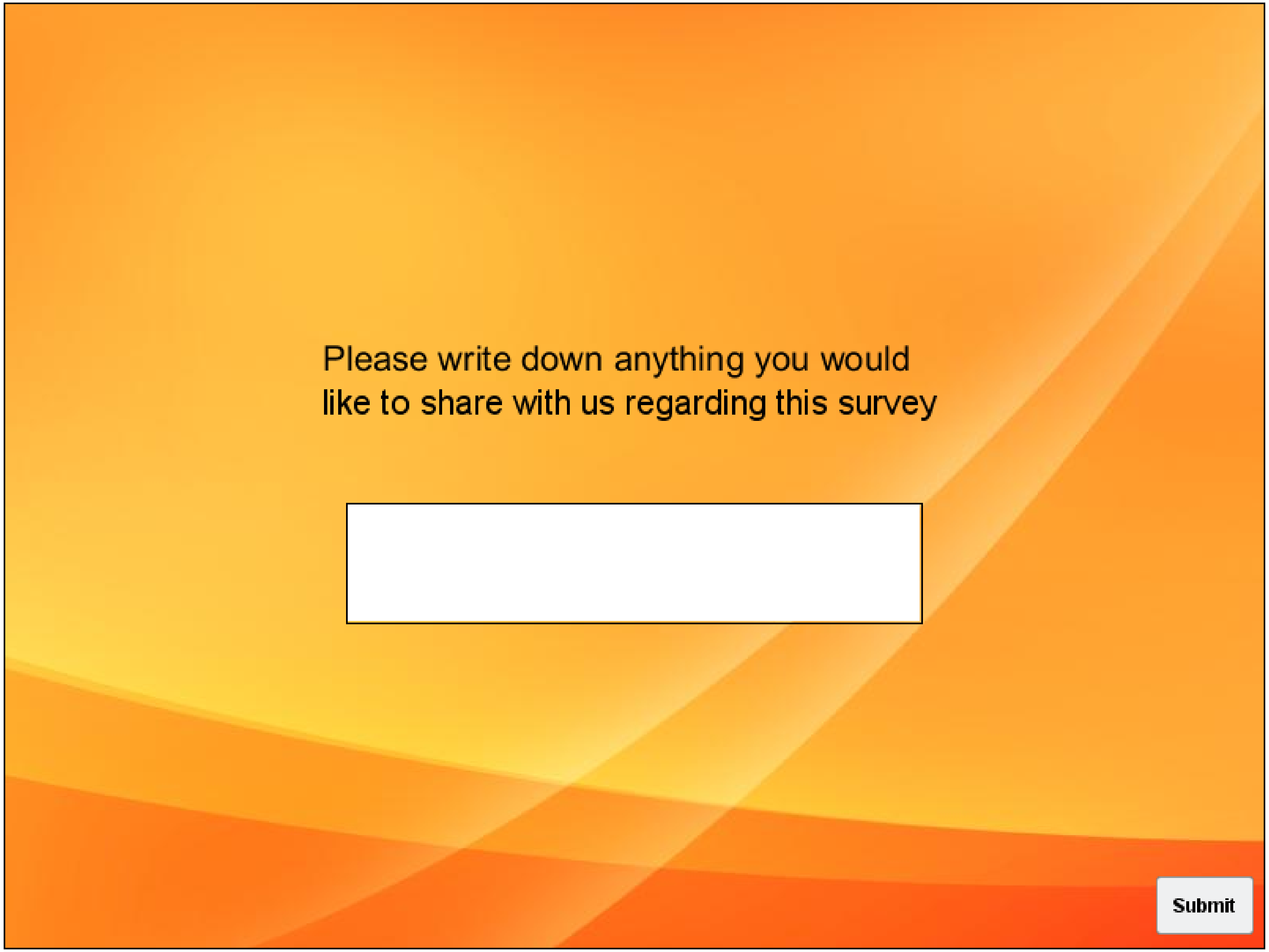
Please write down anything you would
like to share with us regarding this survey
Submit

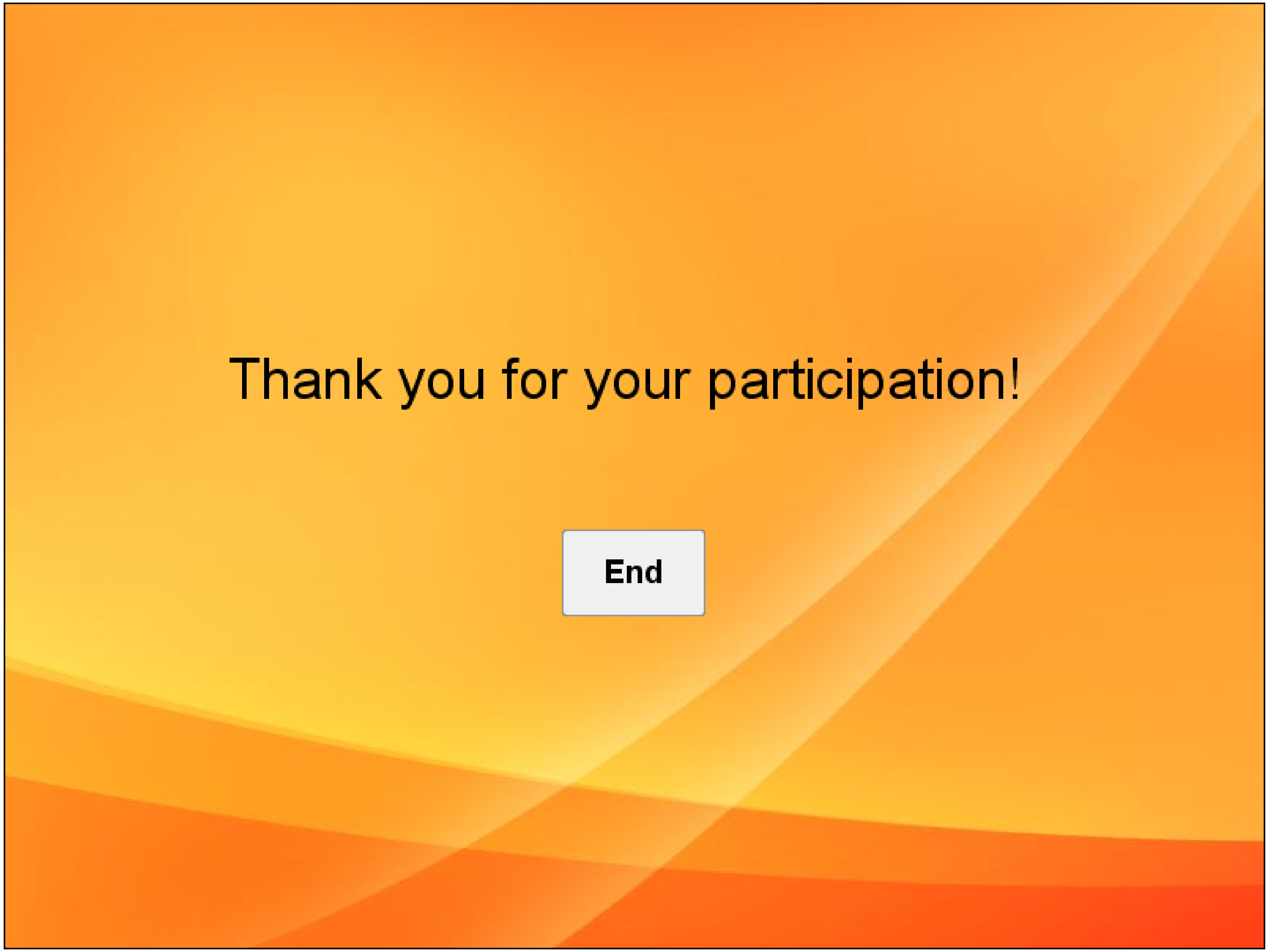
Thank you for your participation!
End
