# Supplementary material for: A descriptive system for the Infant health-related Quality of life Instrument (IQI): Measuring health with a mobile app
Source: PLoS One. 2018 Aug 31;13(8):e0203276. doi: 10.1371/journal.pone.0203276 (PMC6118381; doi:10.1371/journal.pone.0203276)

Introductory Slide

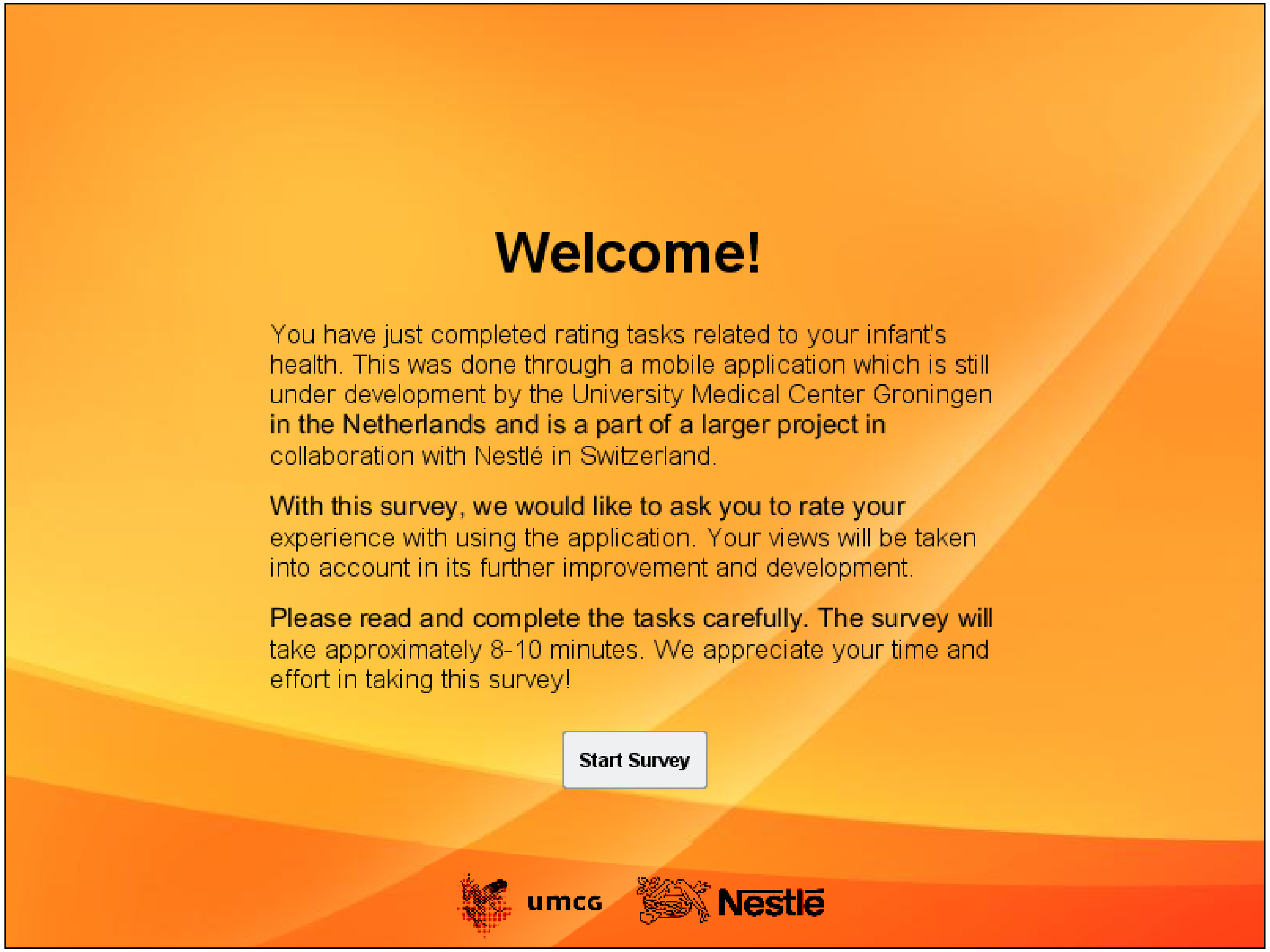

Welcome screen

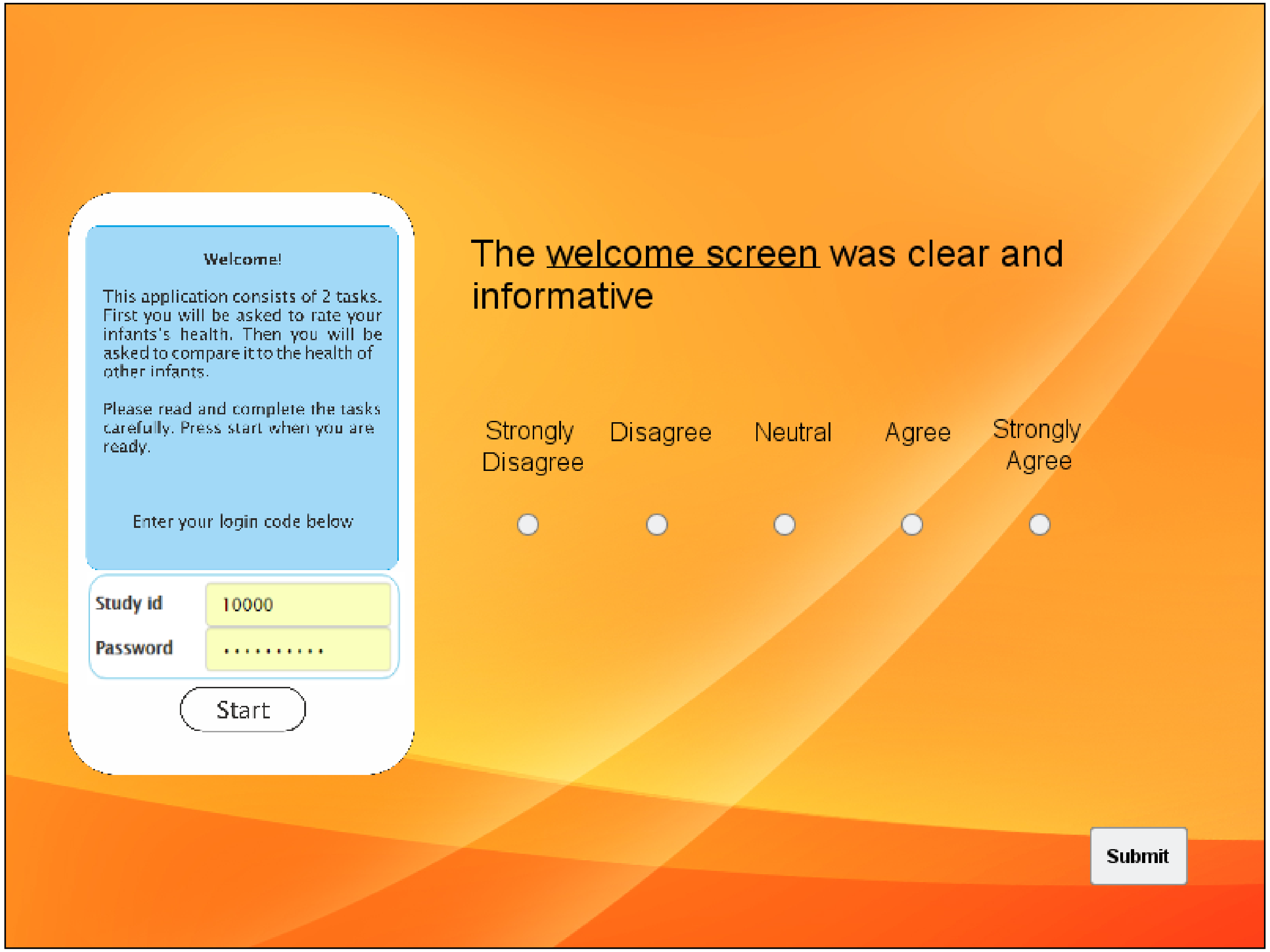

Start task 1

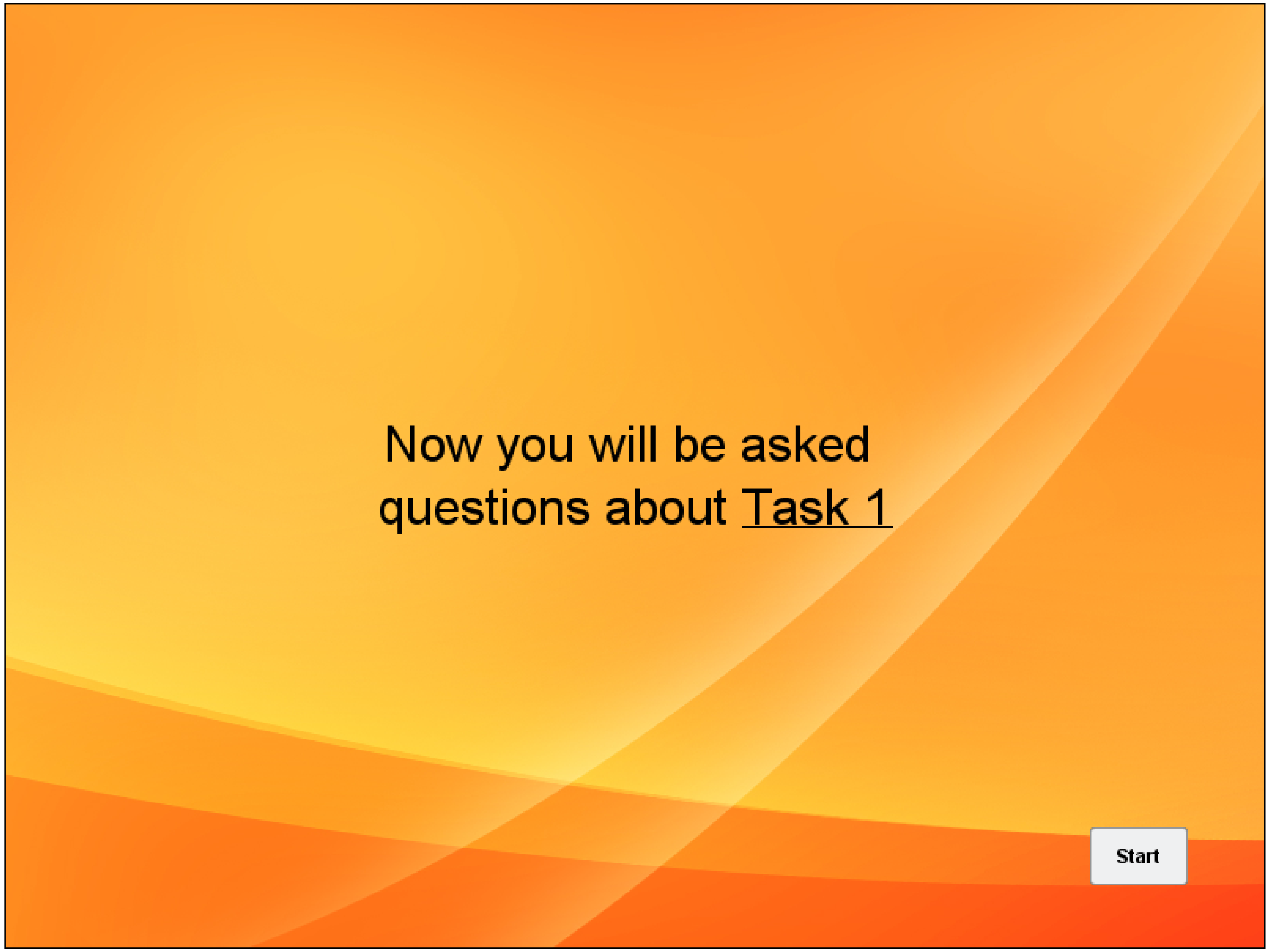

Task 1 instr.

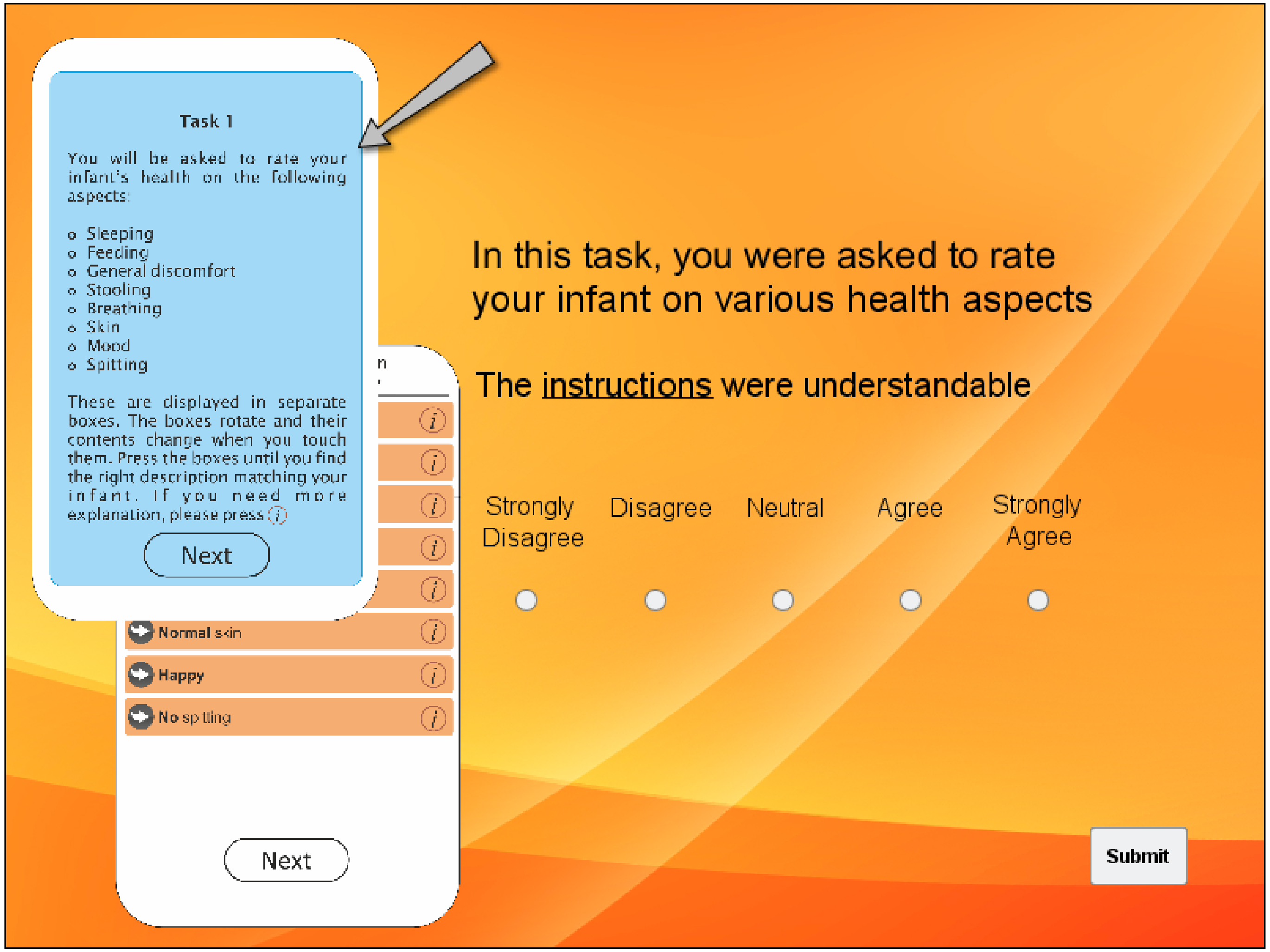
Task 1
You will be asked to rate your infant's health on the following aspects:
Sleeping
Feeding
General discomfort
Stooling
Breathing
Skin
Mood
Spitting
These are displayed in separate boxes. The boxes rotate and their contents change when you touch them. Press the boxes until you find the right description matching your infant. If you need more explanation, please press
Next
Normal skin
Happy
No spitting
Next
In this task, you were asked to rate your infant on various health aspects
The instructions were understandable
Strongly Disagree
Disagree
Neutral
Agree
Strongly Agree
Submit

Task 1 rotation

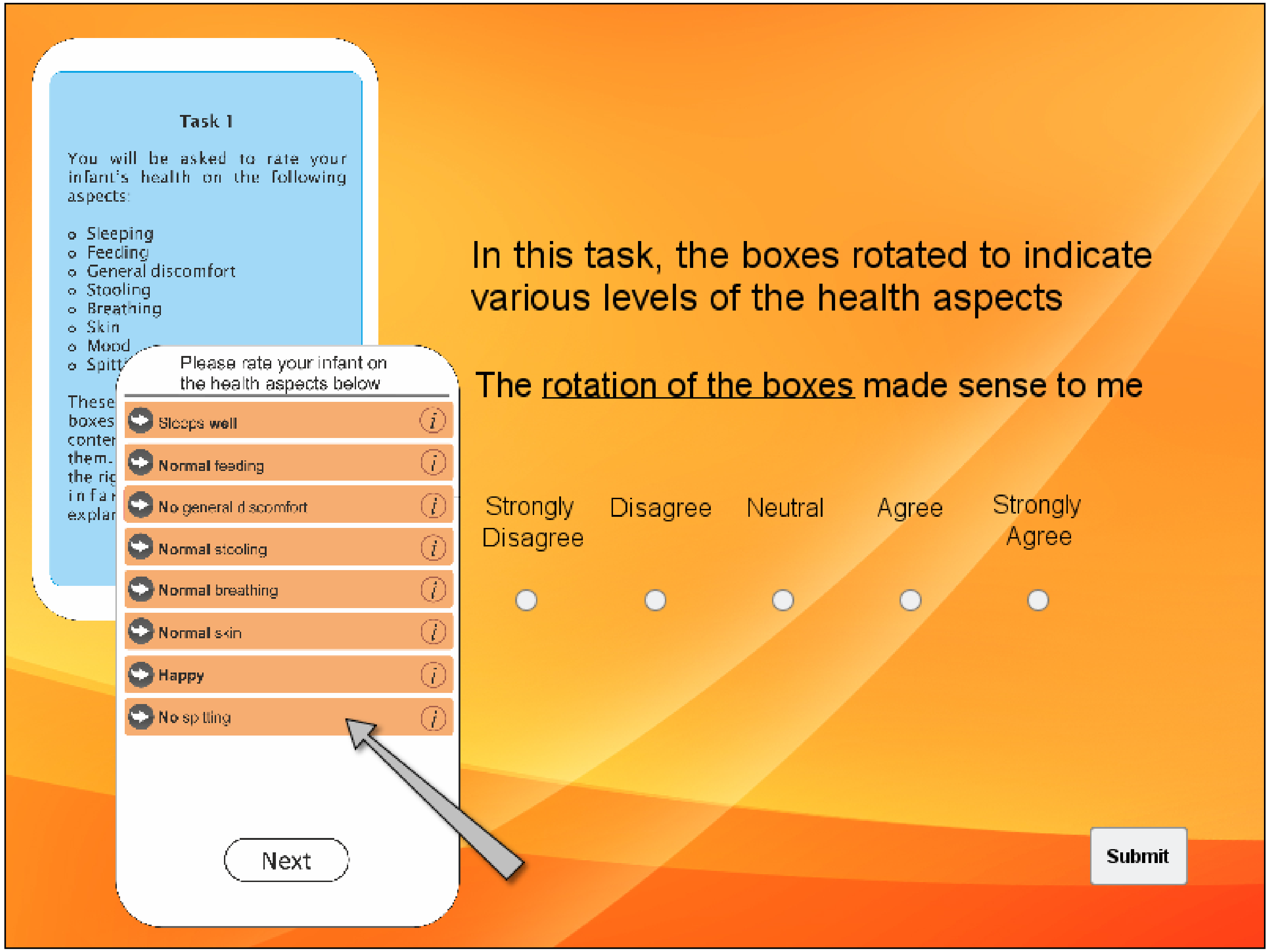

Task 1 rotation

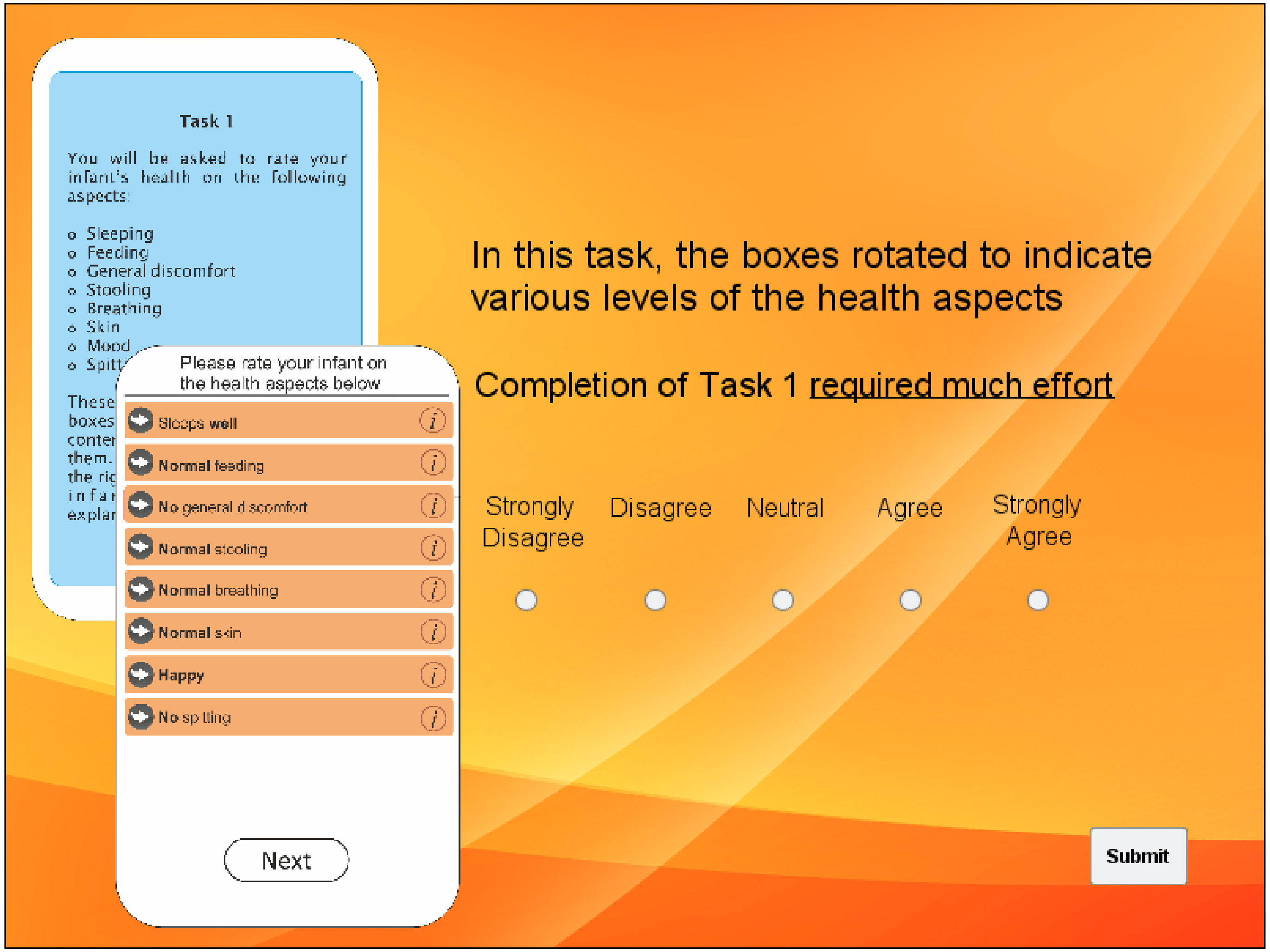

Task 1 help screen making use

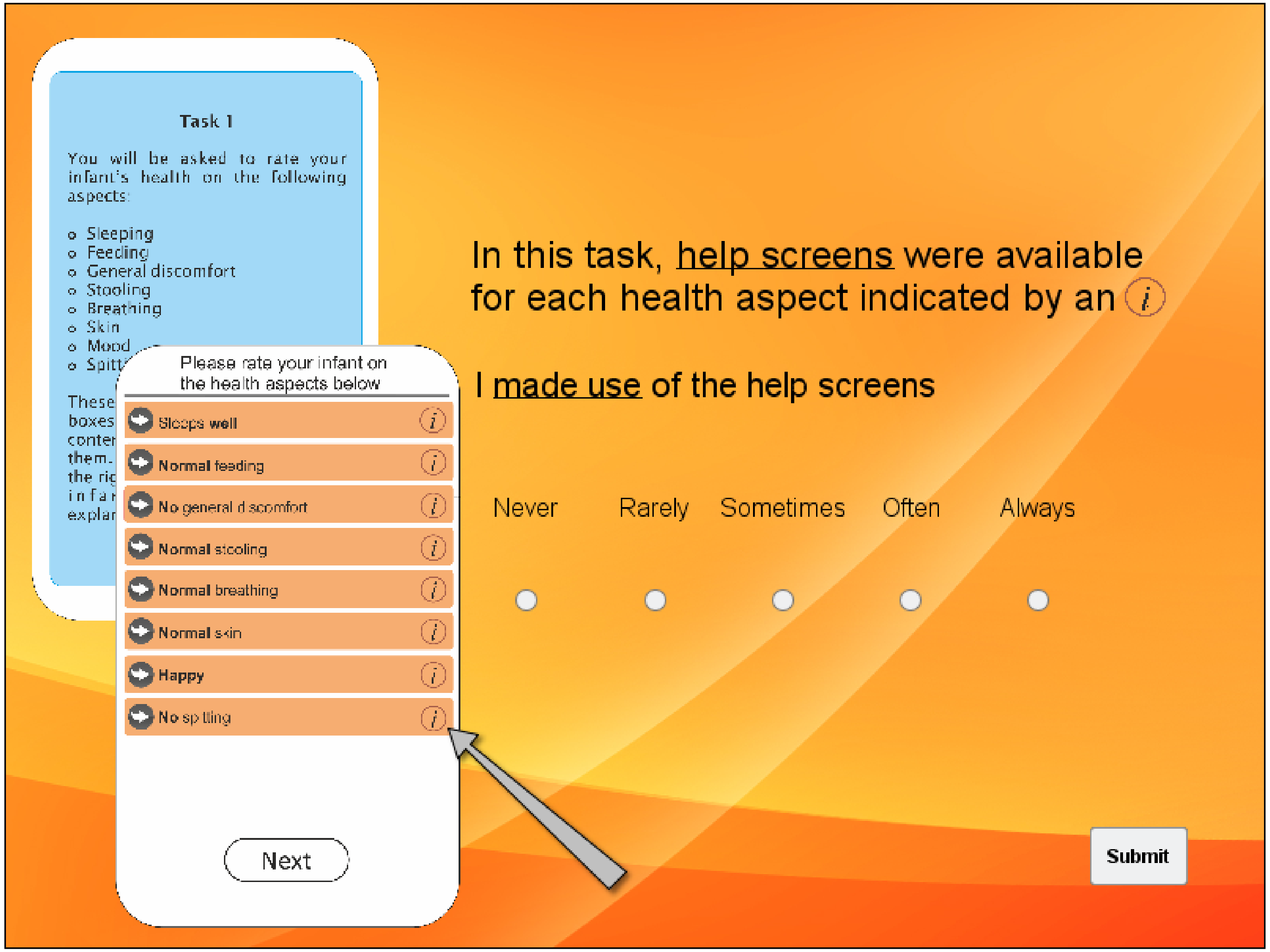

Task 1 help screen usefulness

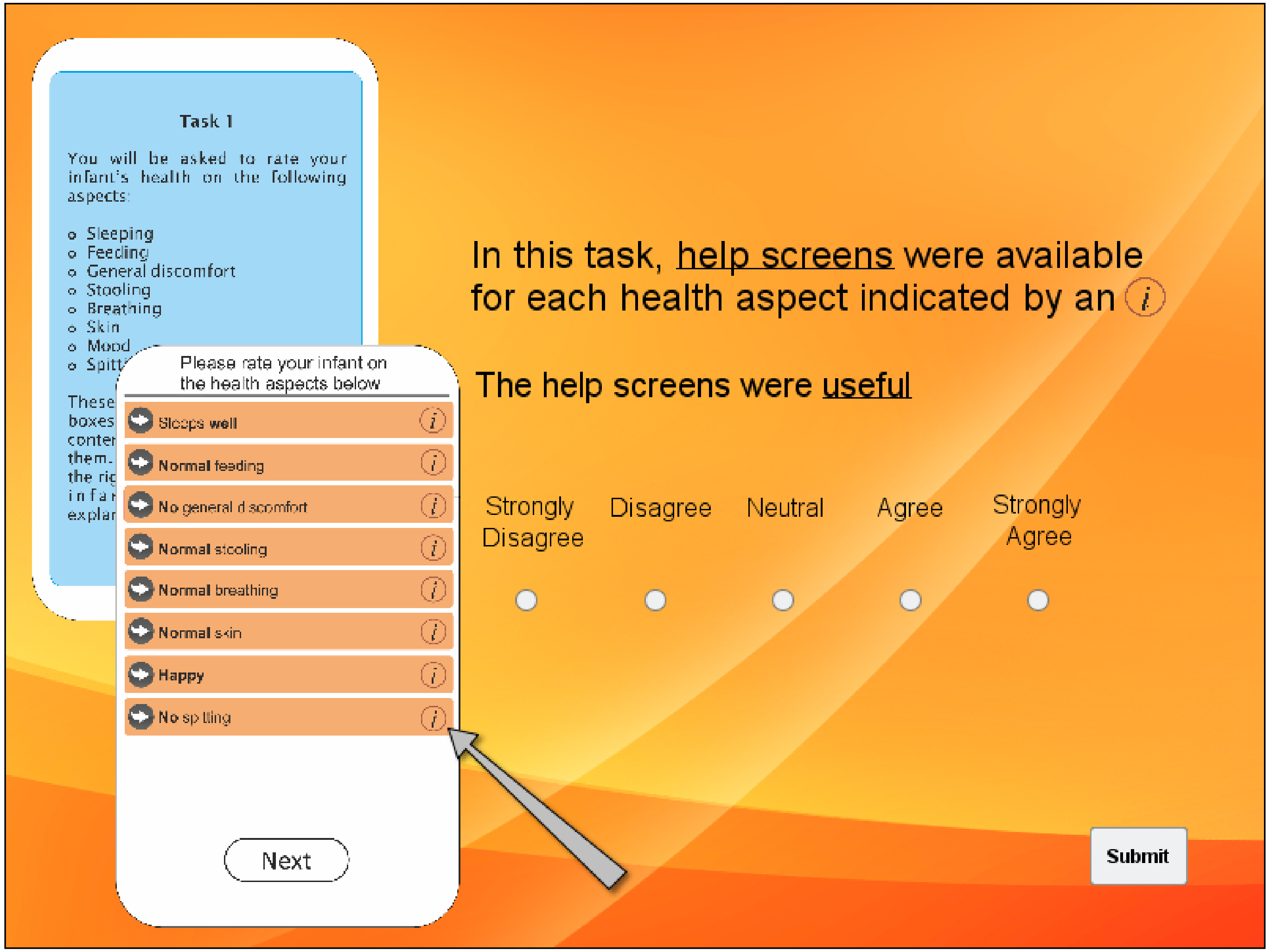

Start help screens

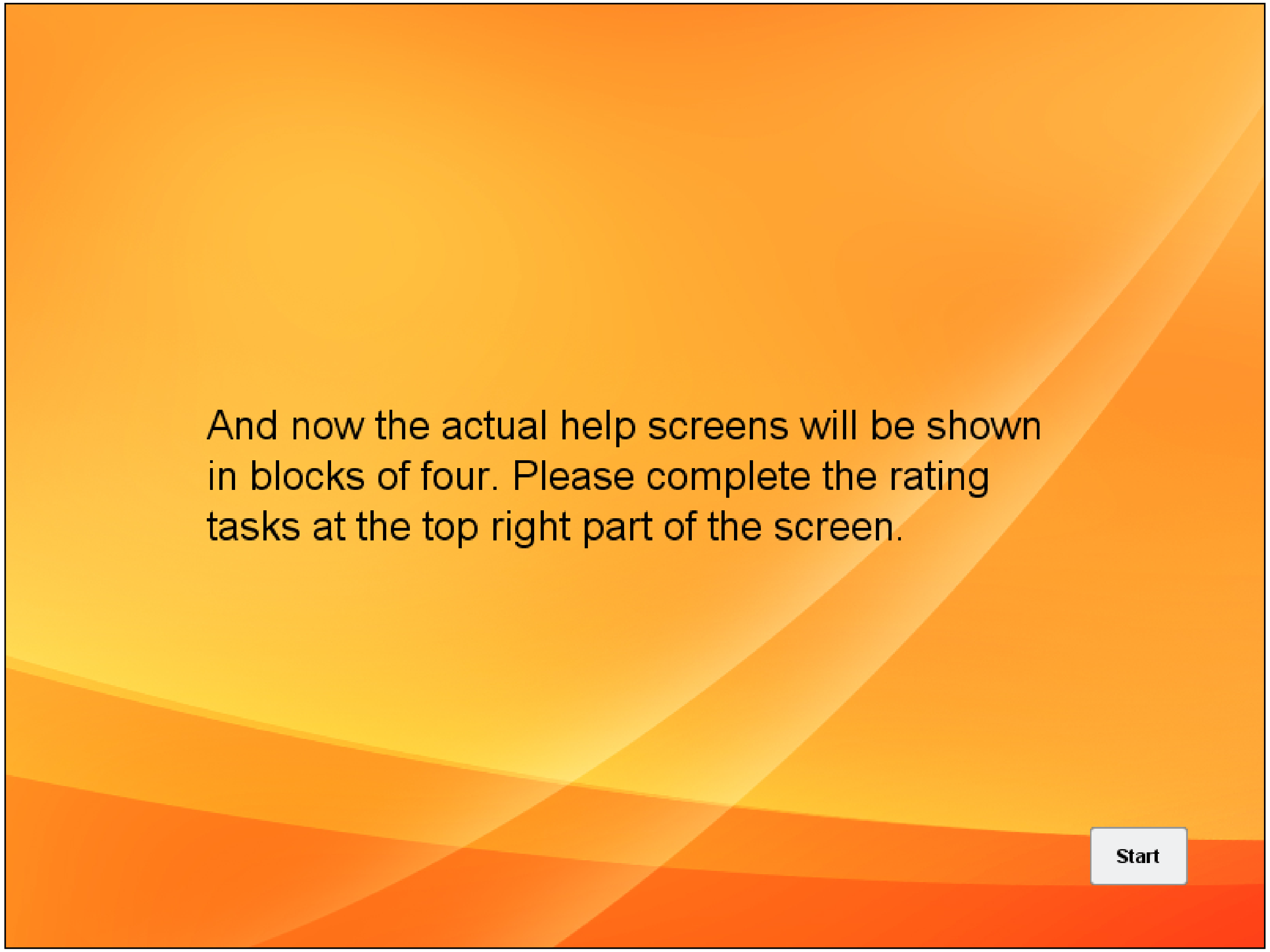

Help screen block 1 understanding

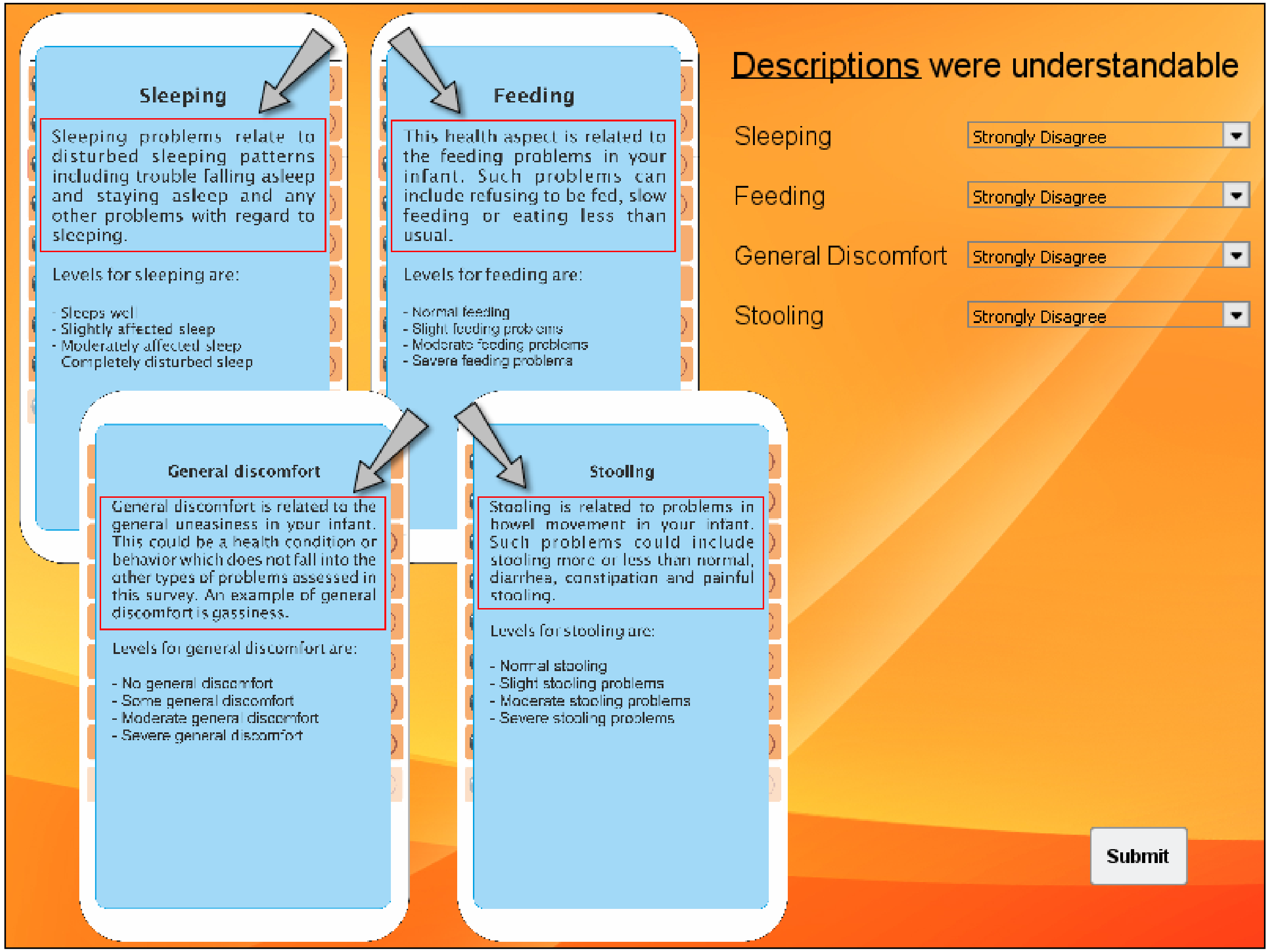

Help screen block 1 meaningfulness

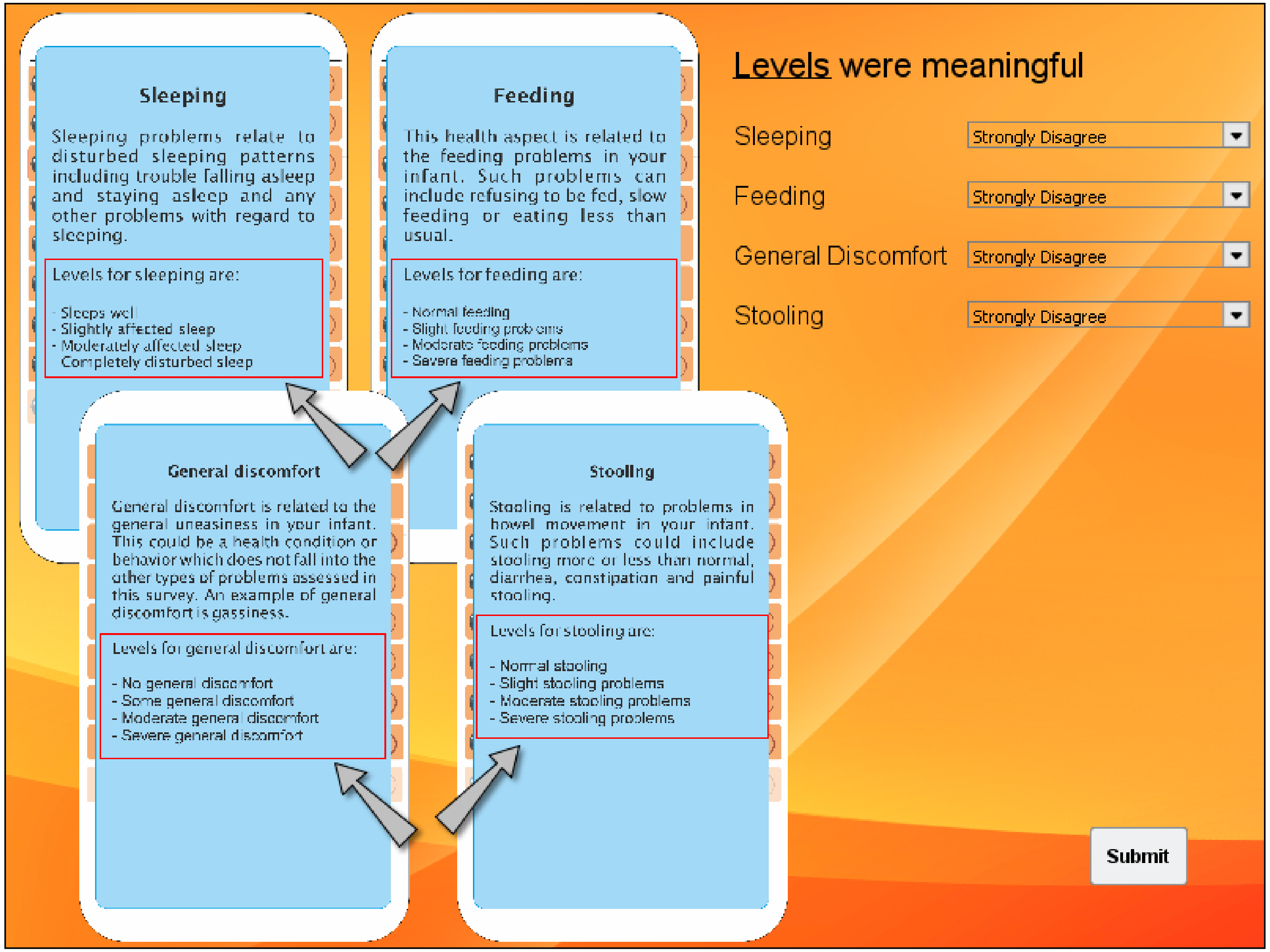

Help screen block 1 open question

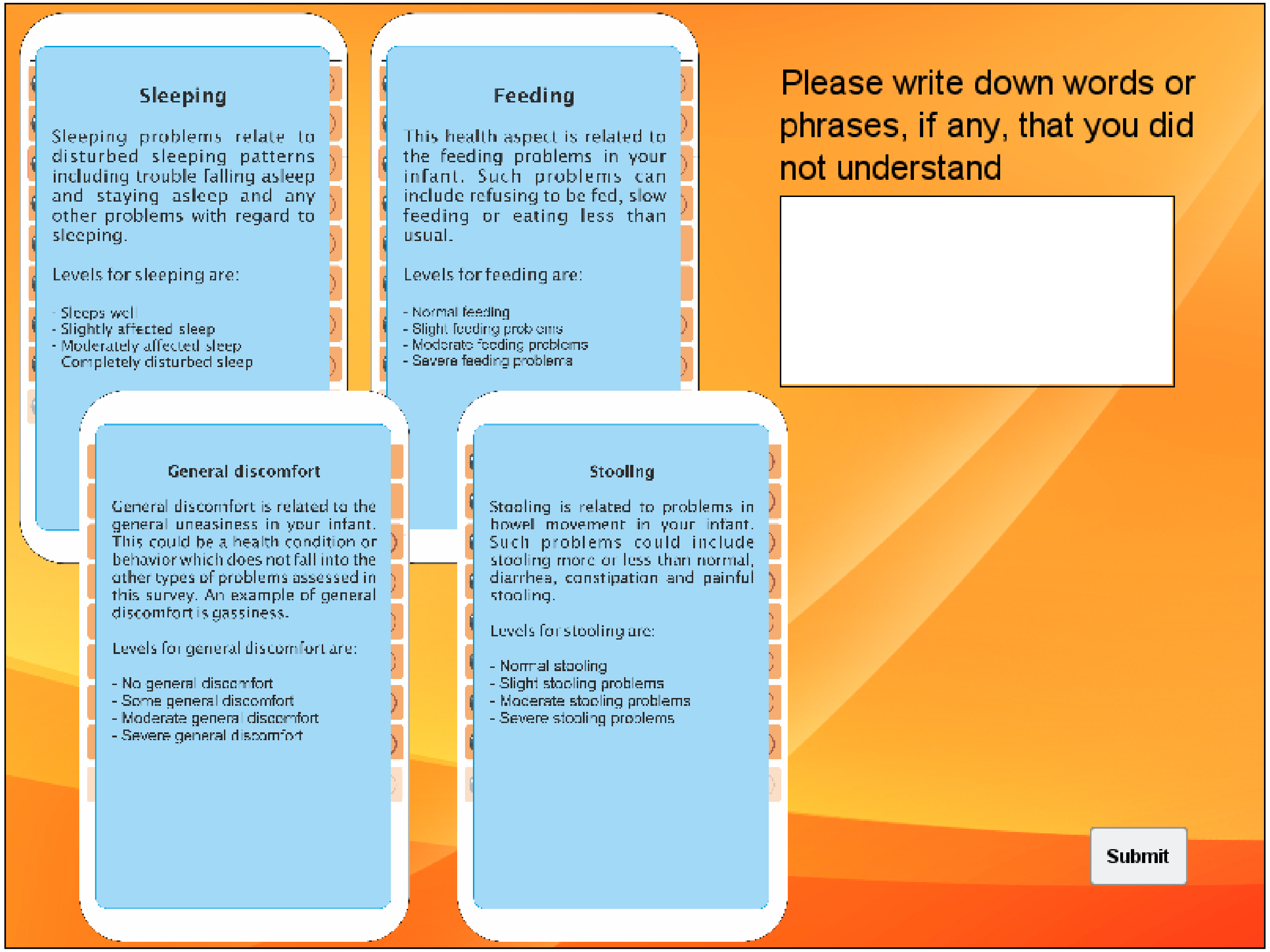

Help screen block 2 understanding

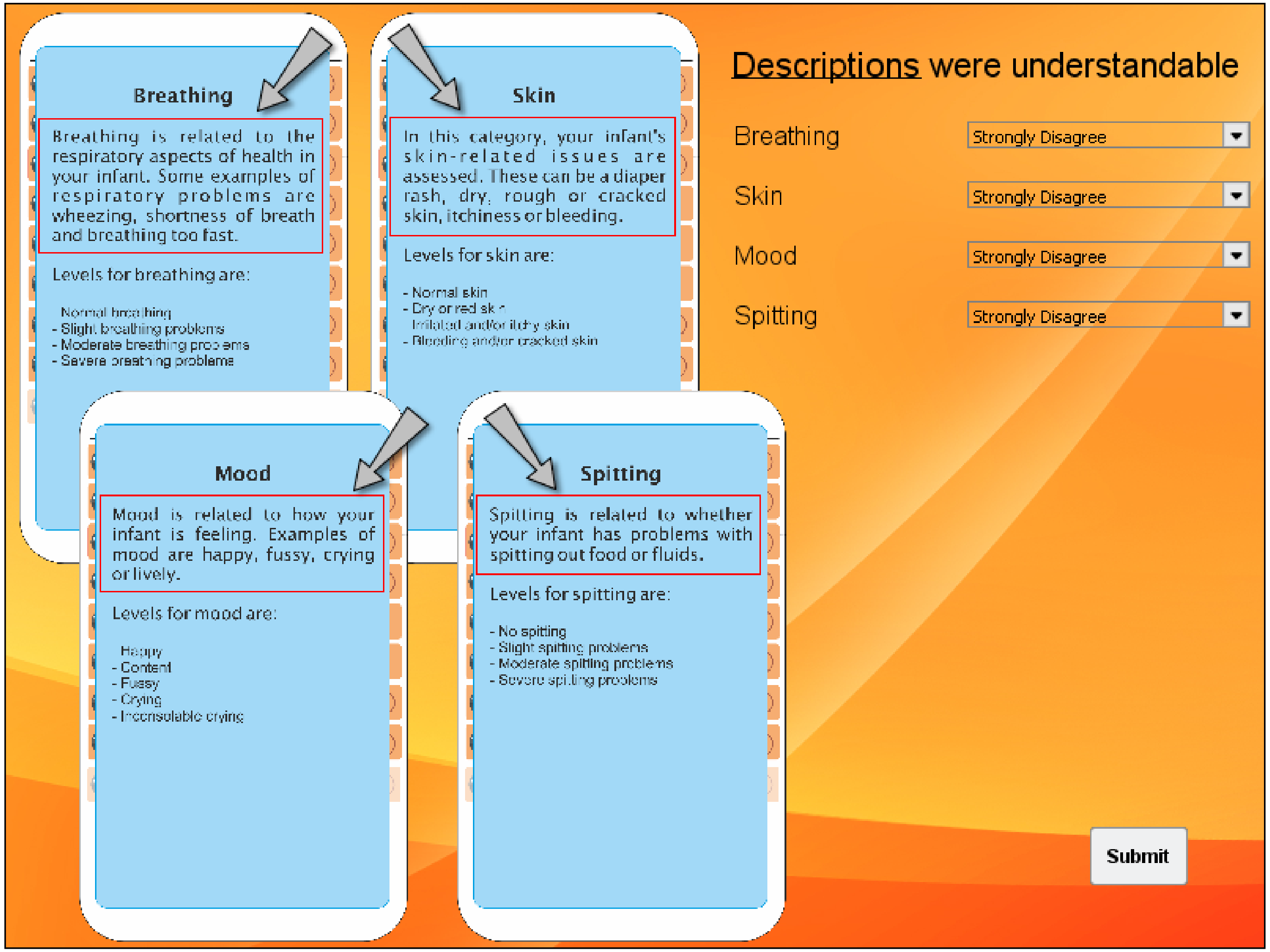

Help screen block 2 meaningfullness

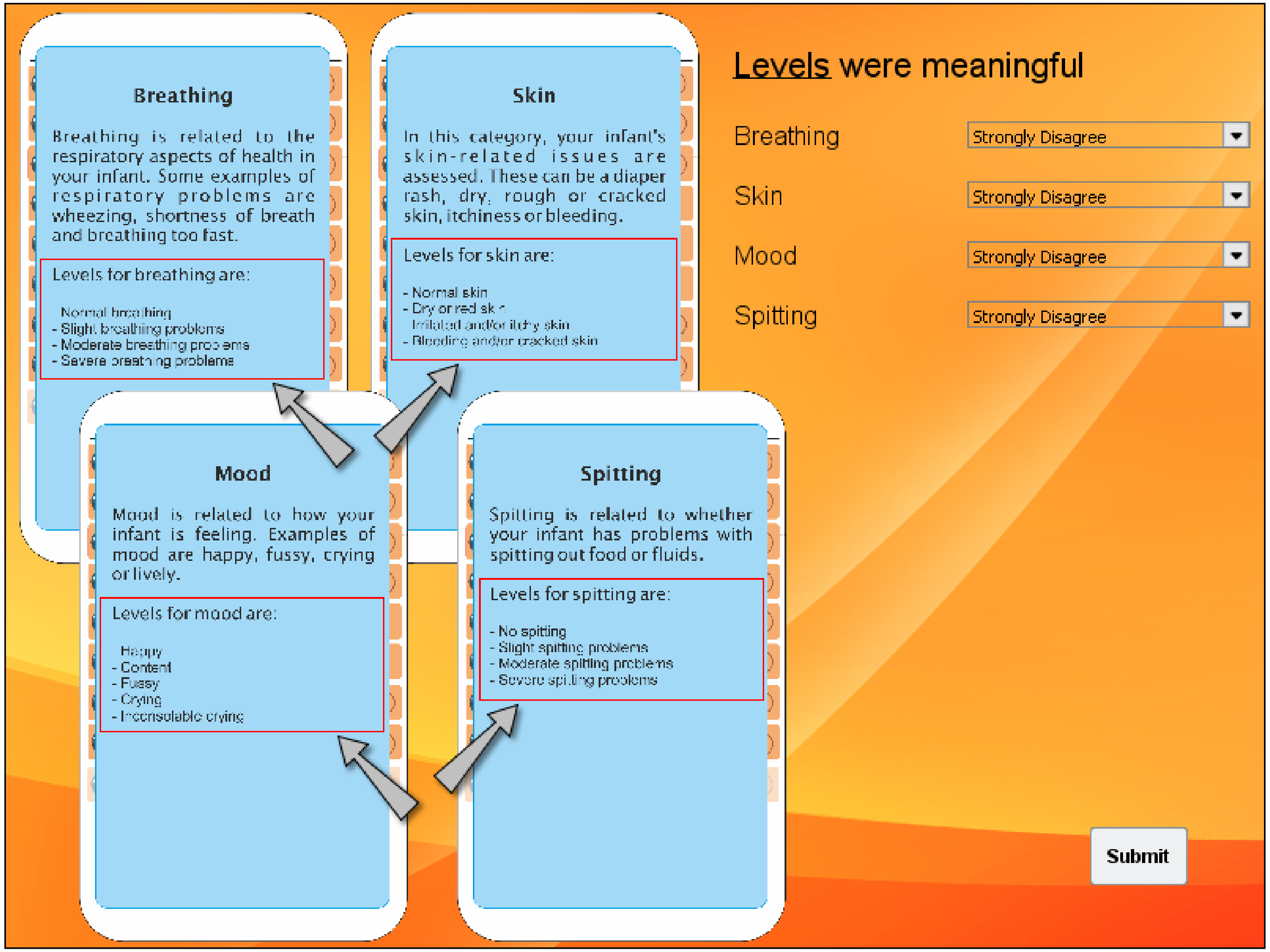

Help screen block 2 open question

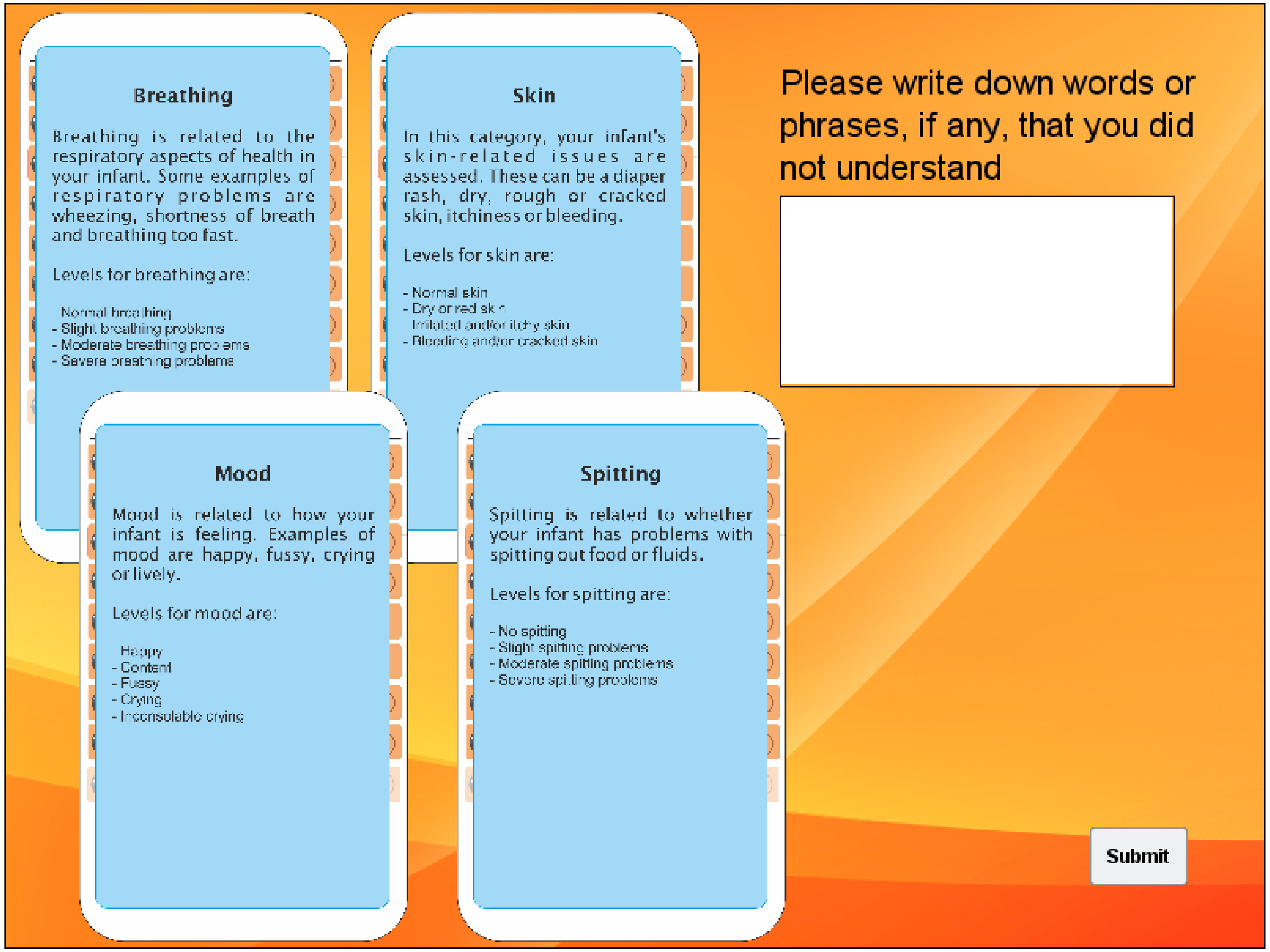

Task 1 open question

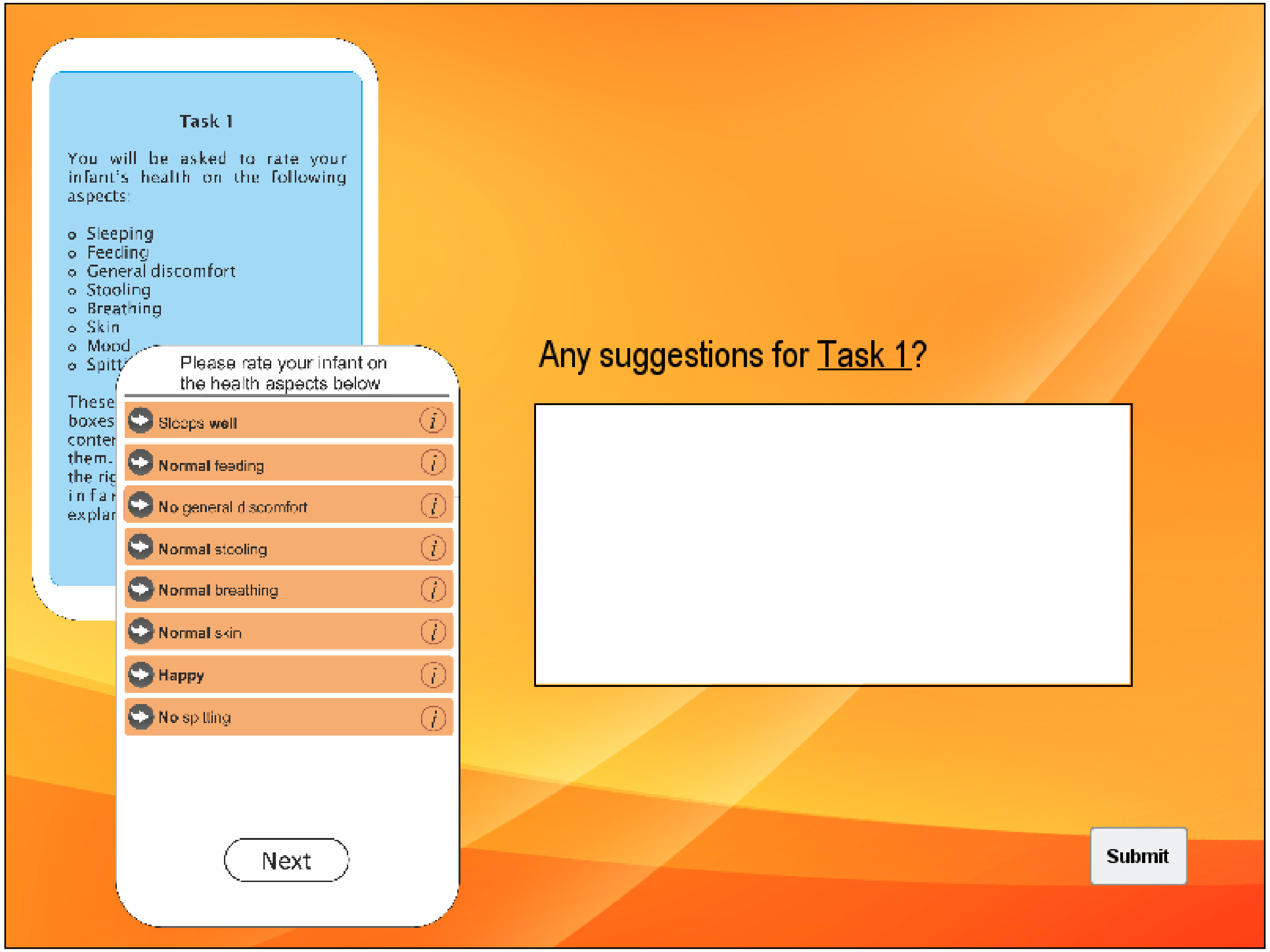

Start task 2

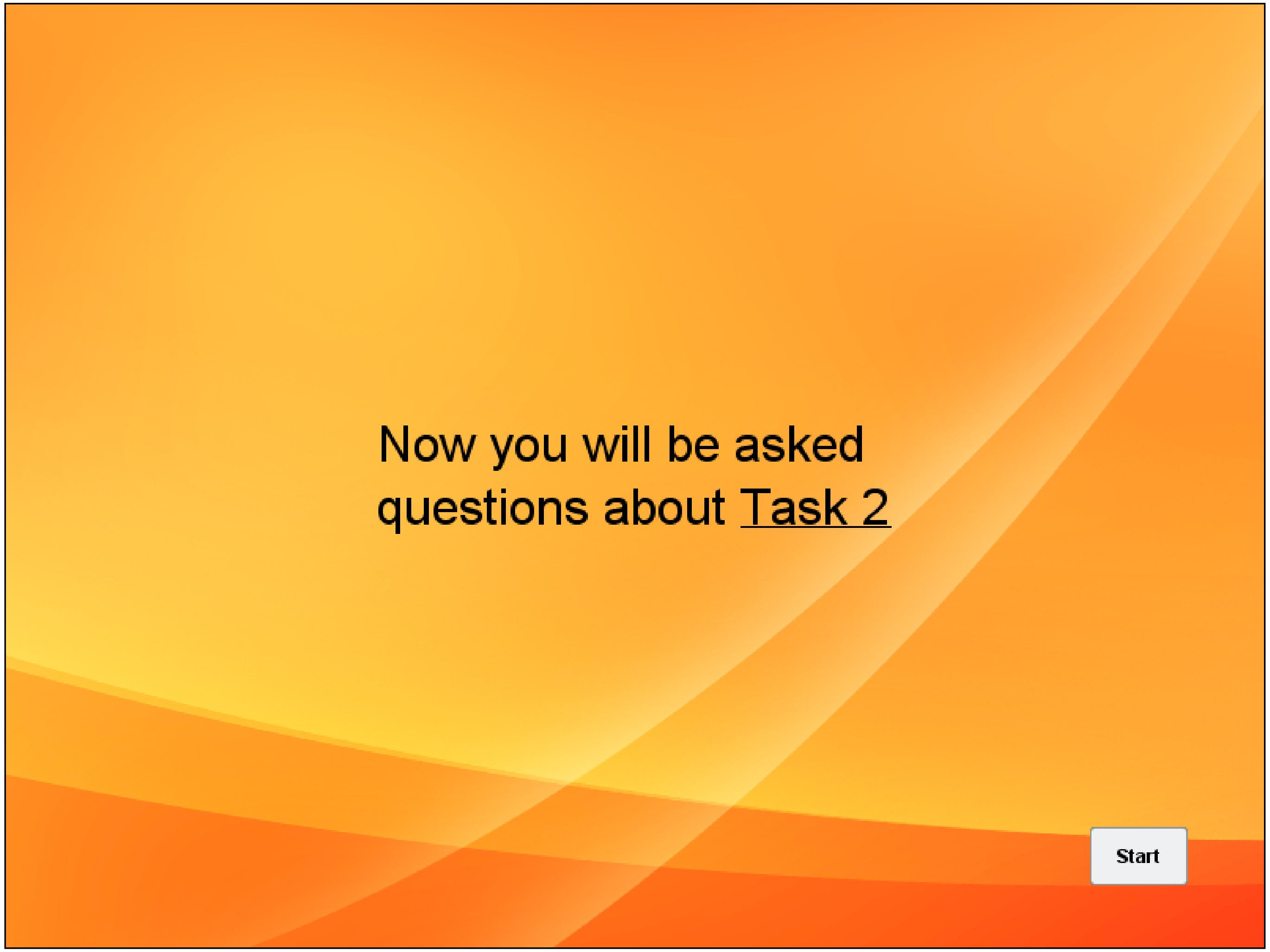

Task 2 instructions

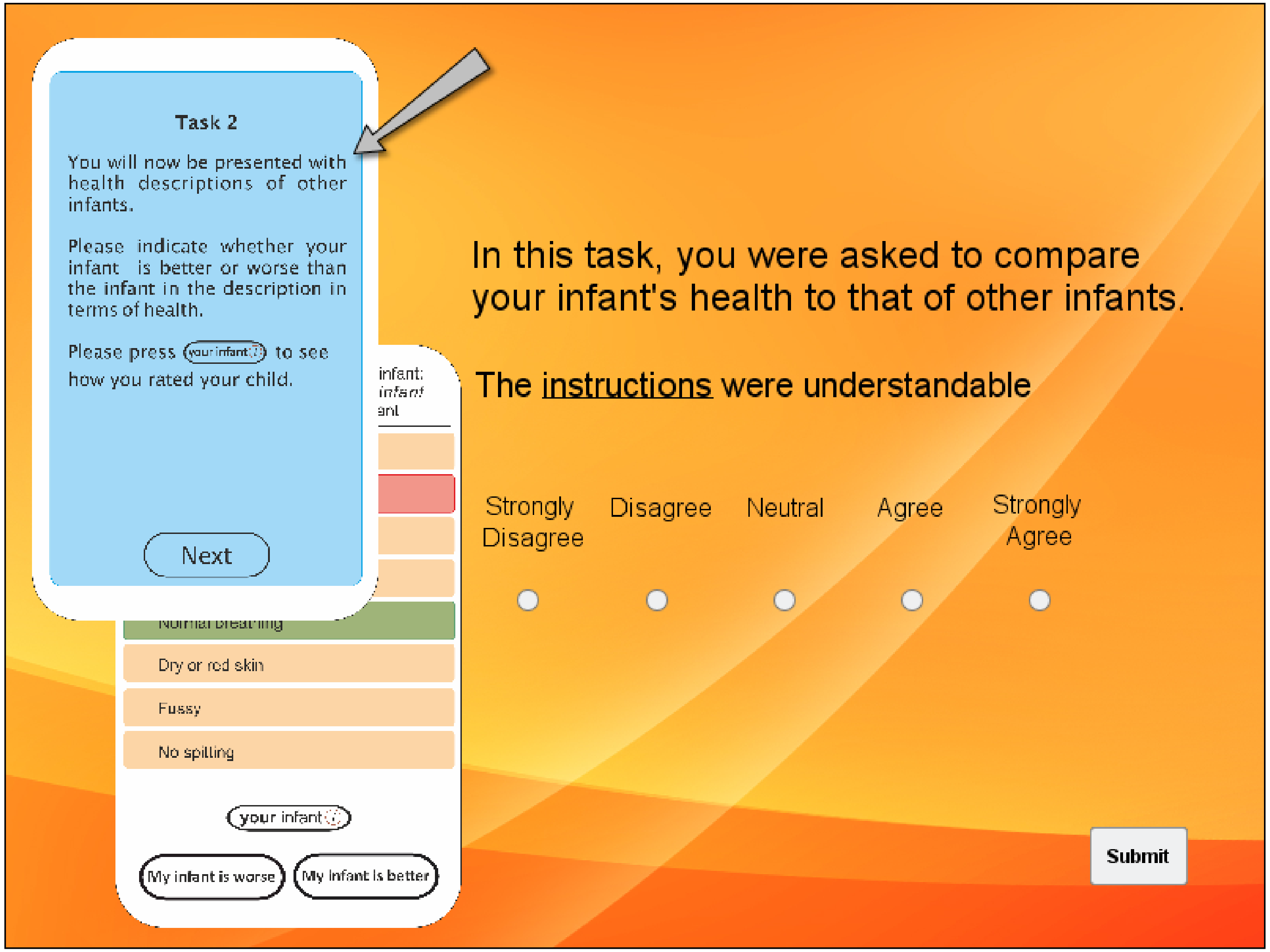

Task 2 red and green color

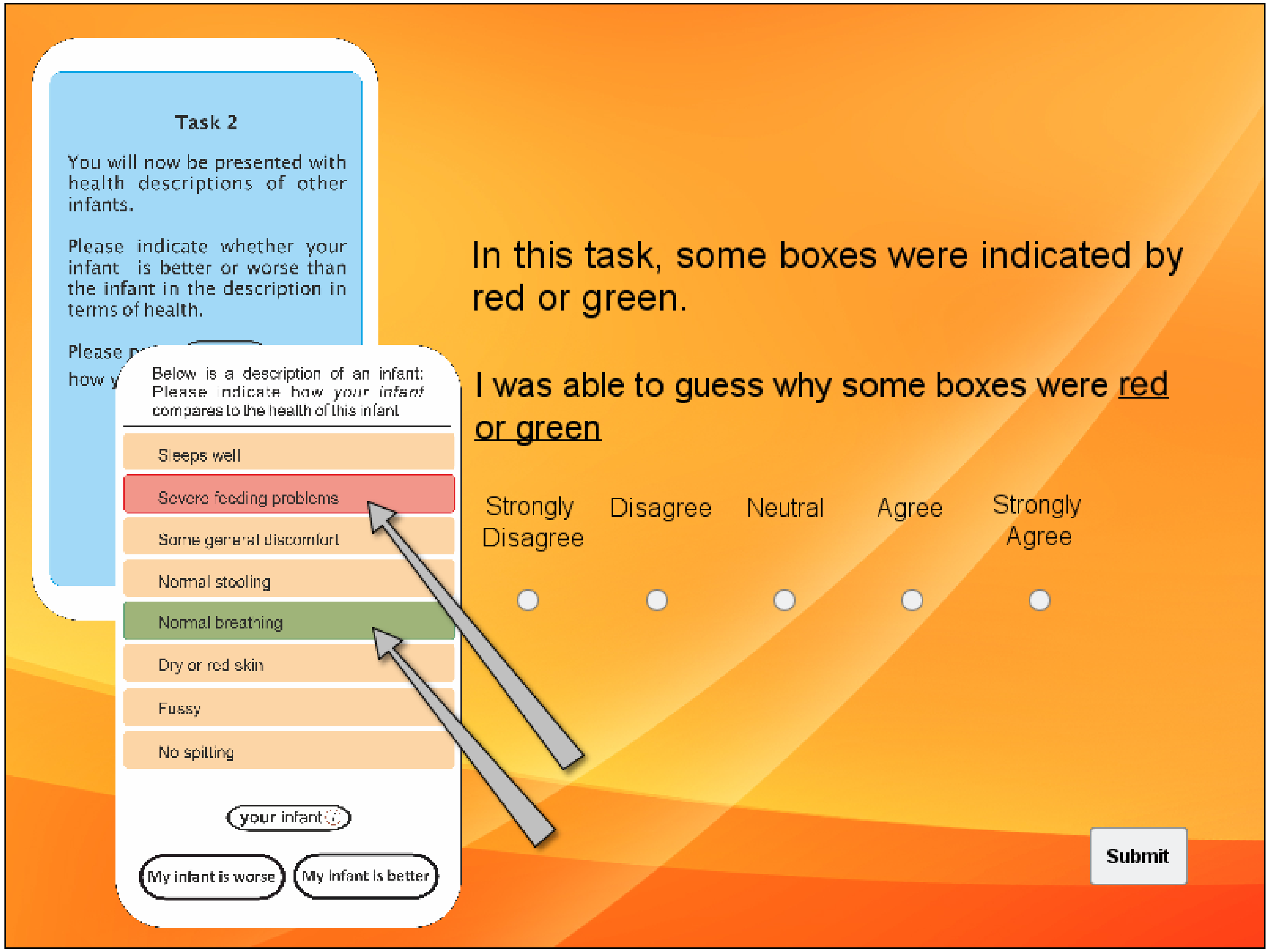

Task 2 my infant

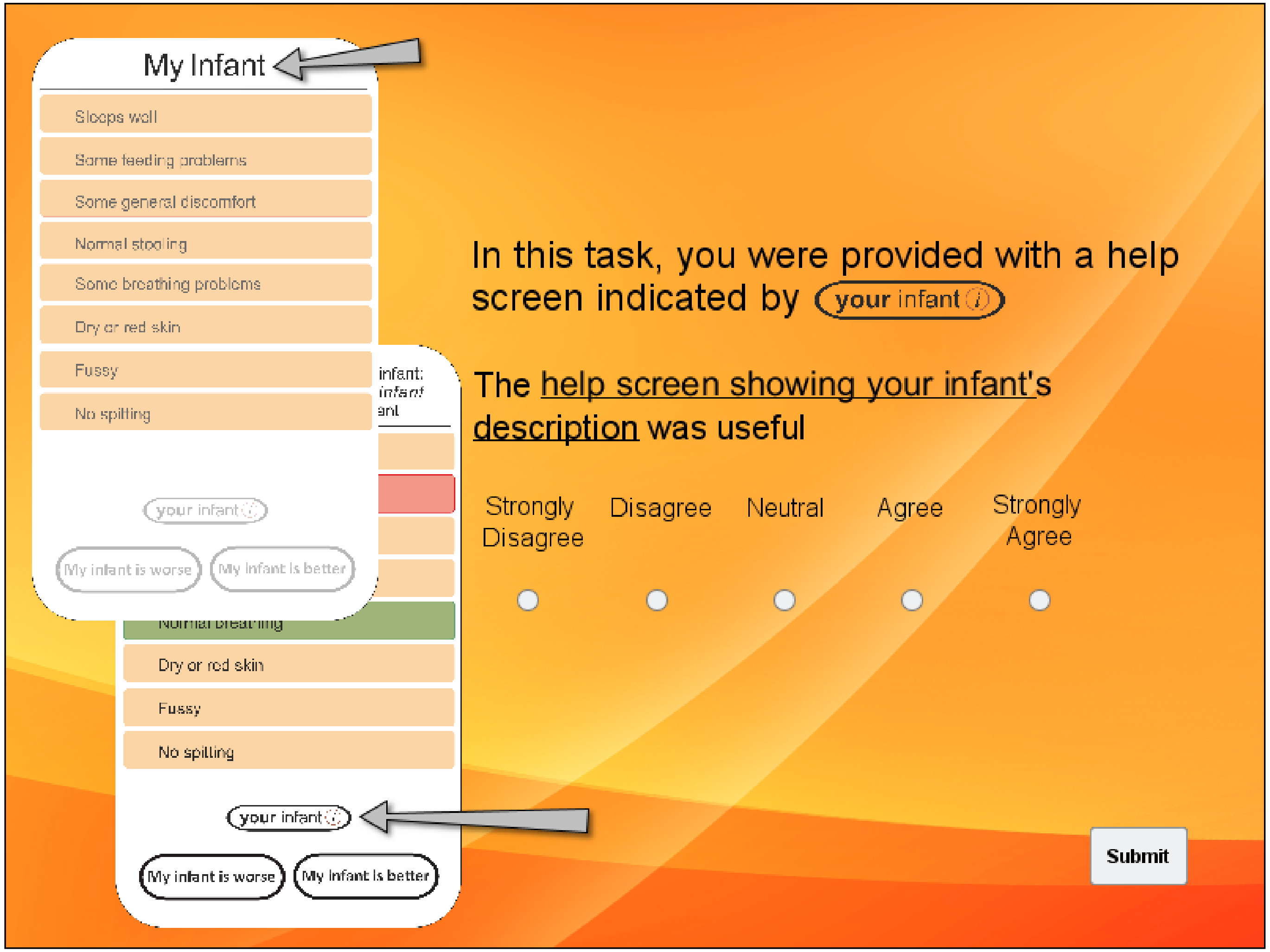

Task 2 open question

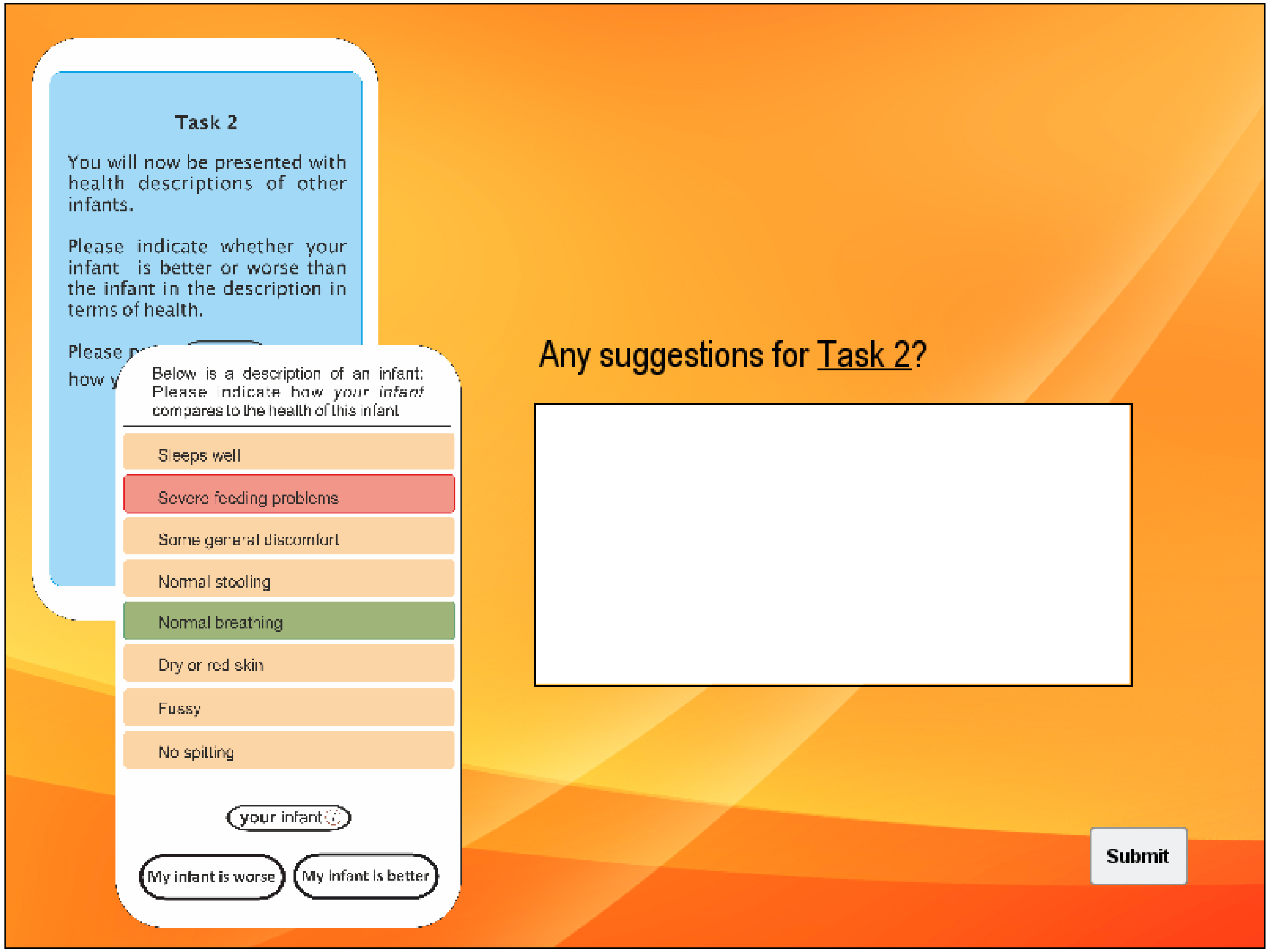

Relevance of the attributes

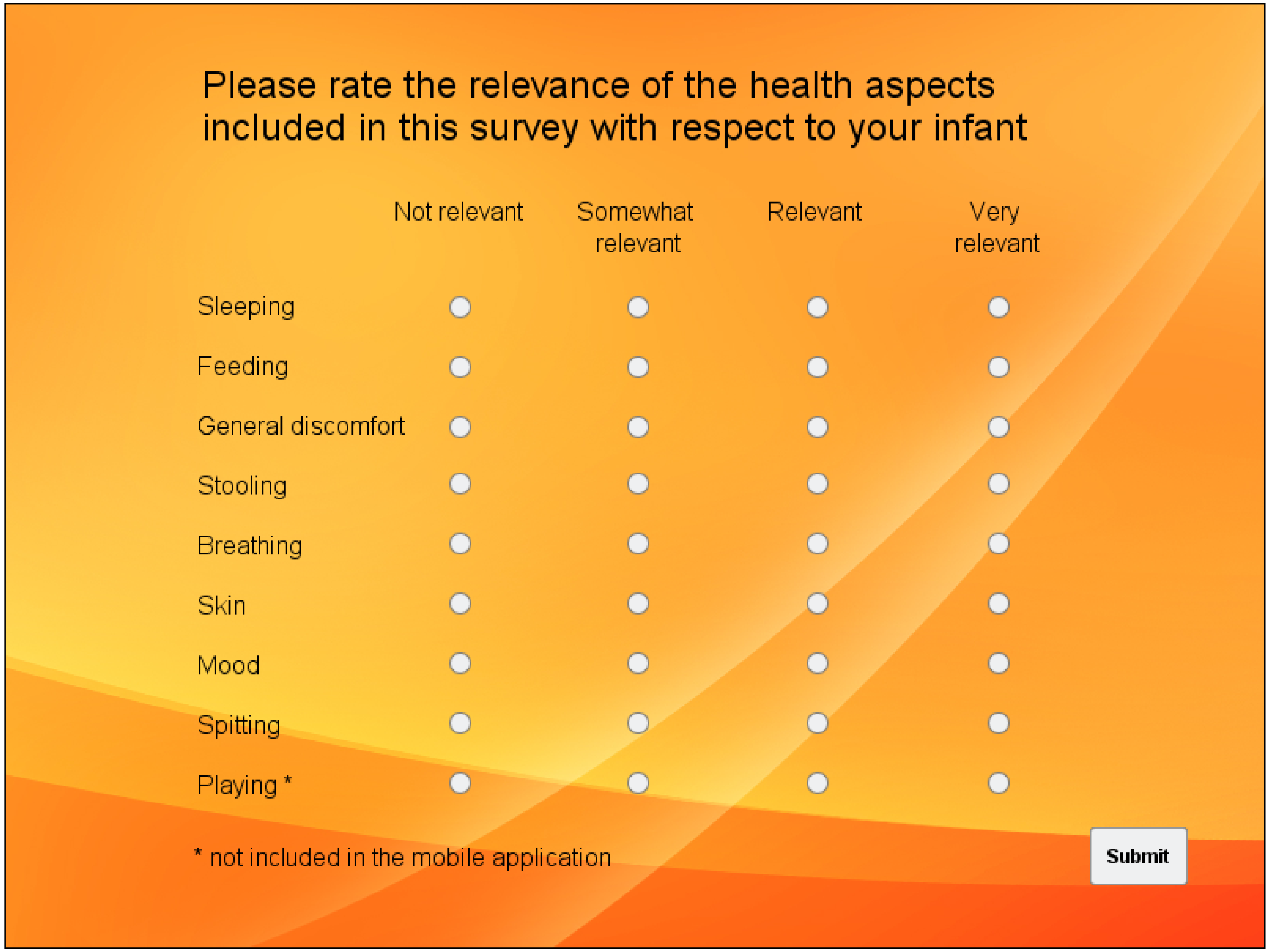

Playing

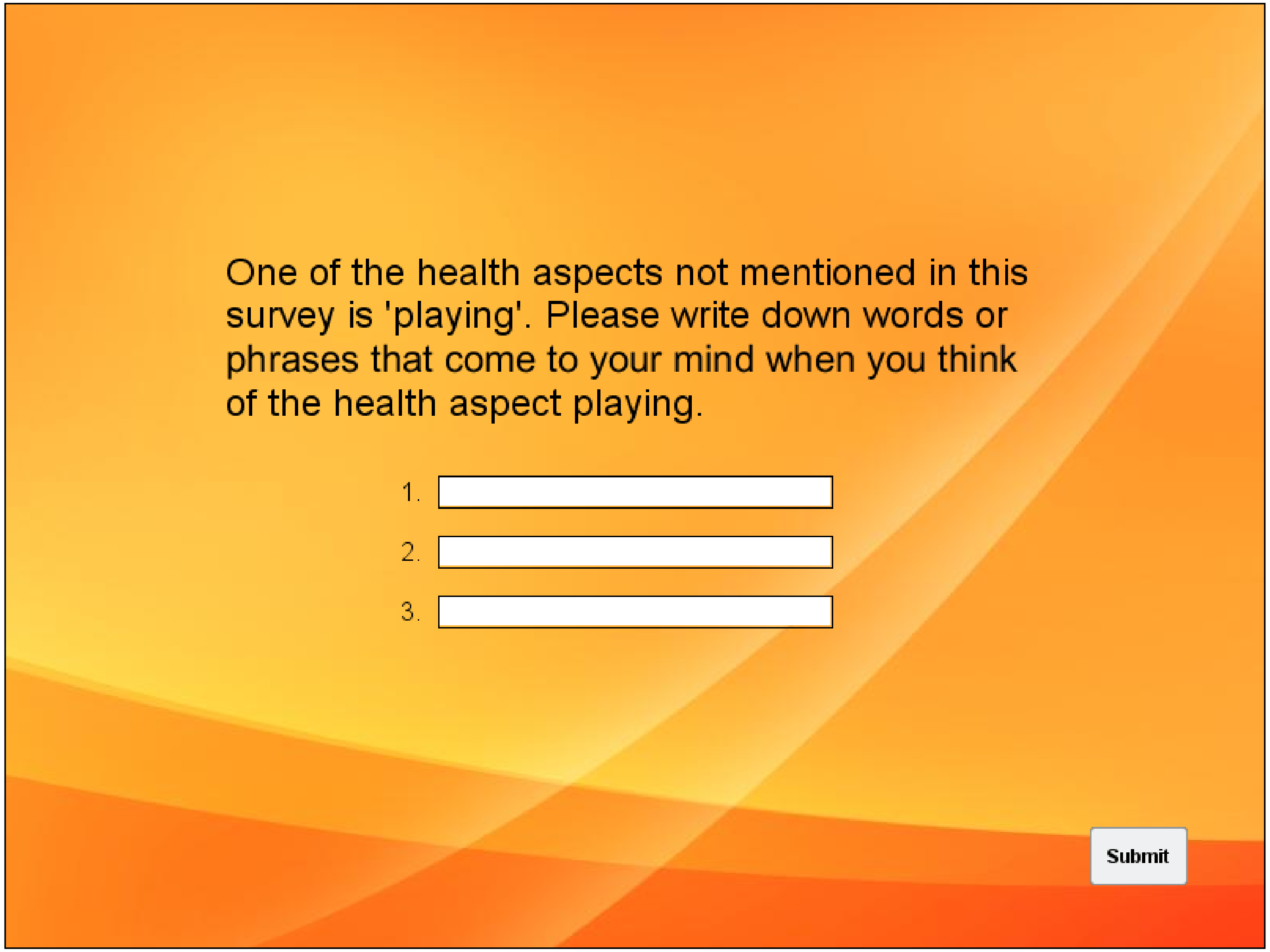
One of the health aspects not mentioned in this survey is 'playing'. Please write down words or phrases that come to your mind when you think of the health aspect playing.
1.
2.
3.
Submit

Task 1 rotation

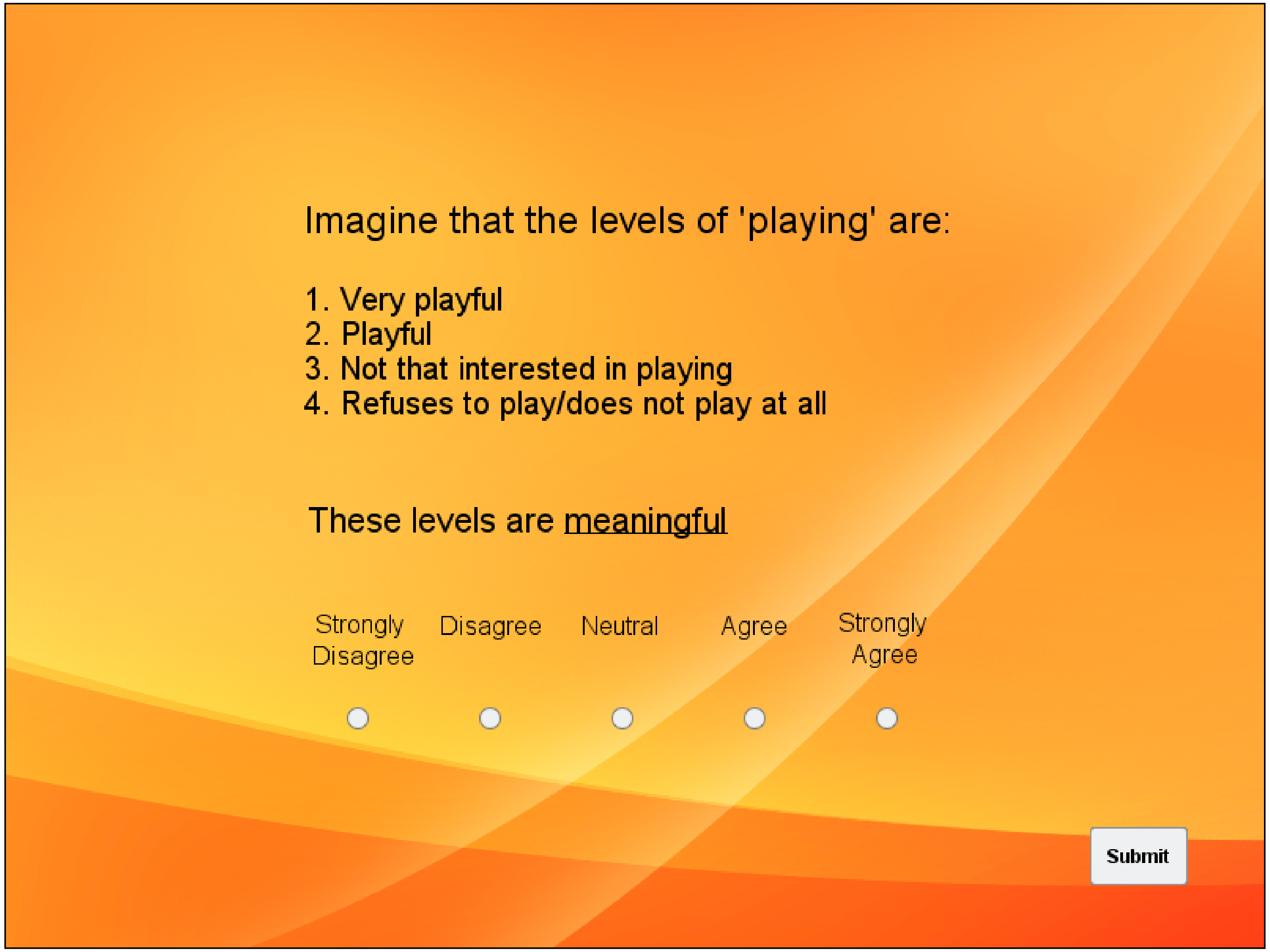

Additional attributes open question

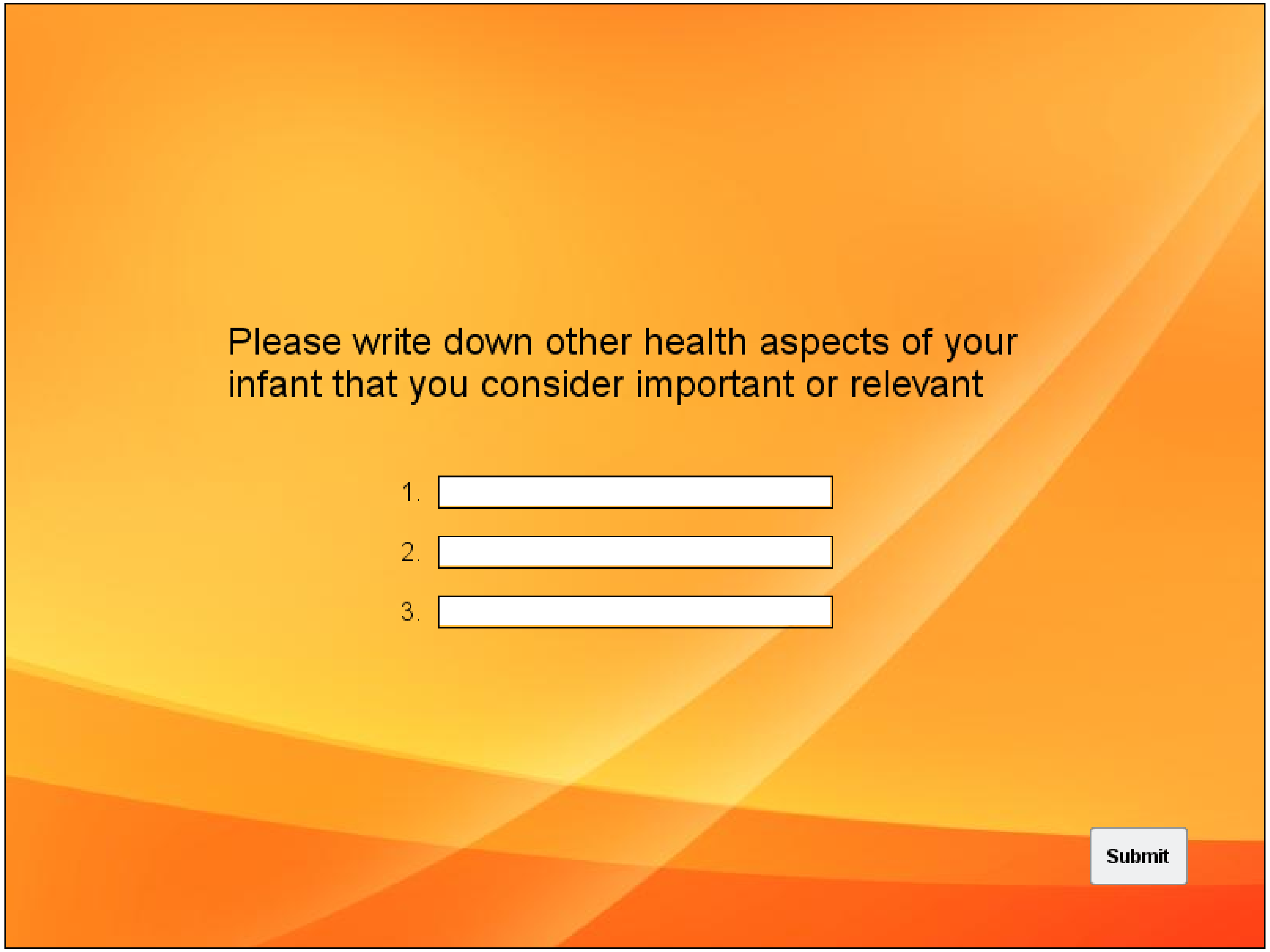

Please indicate

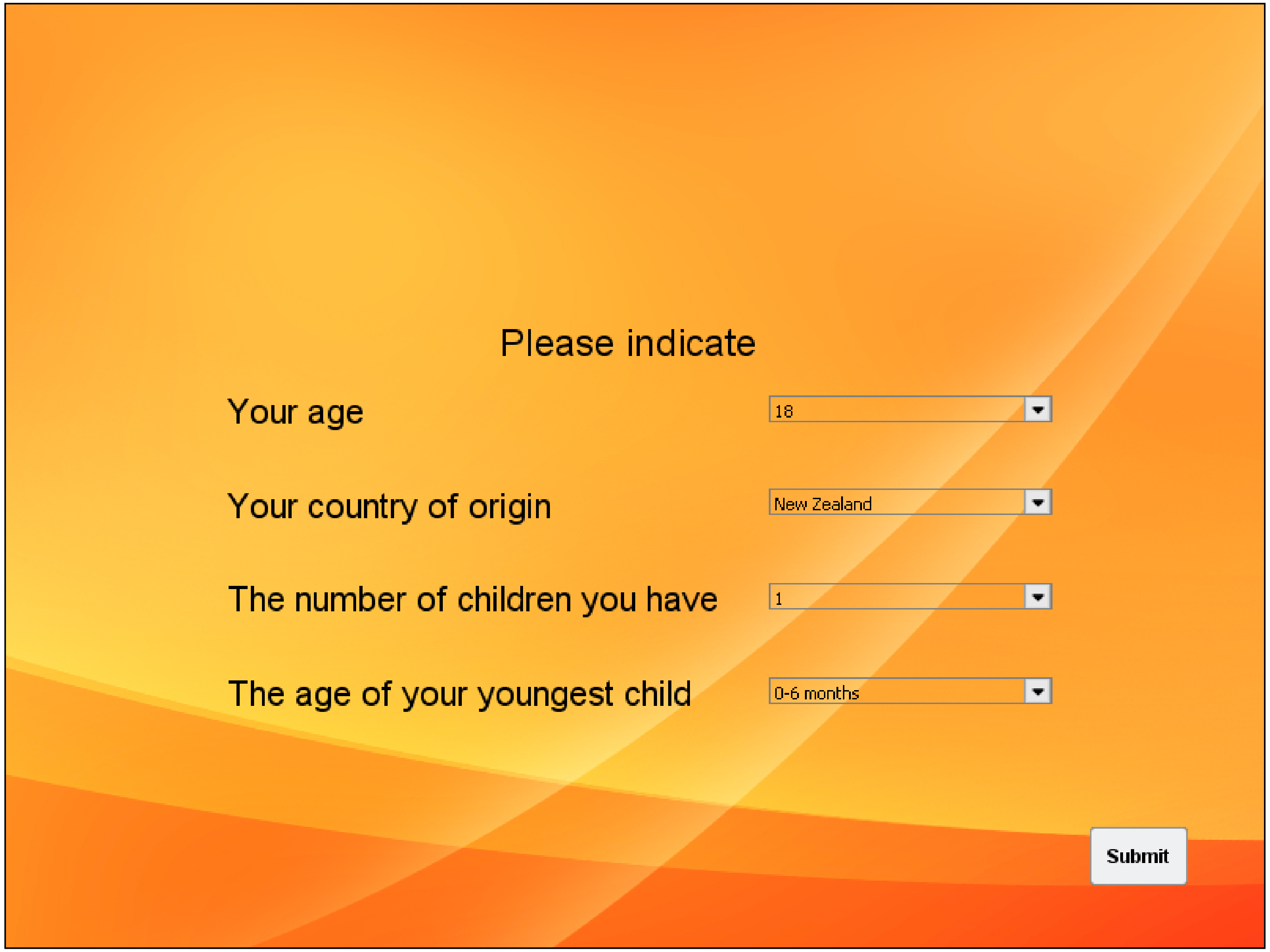

Thank you

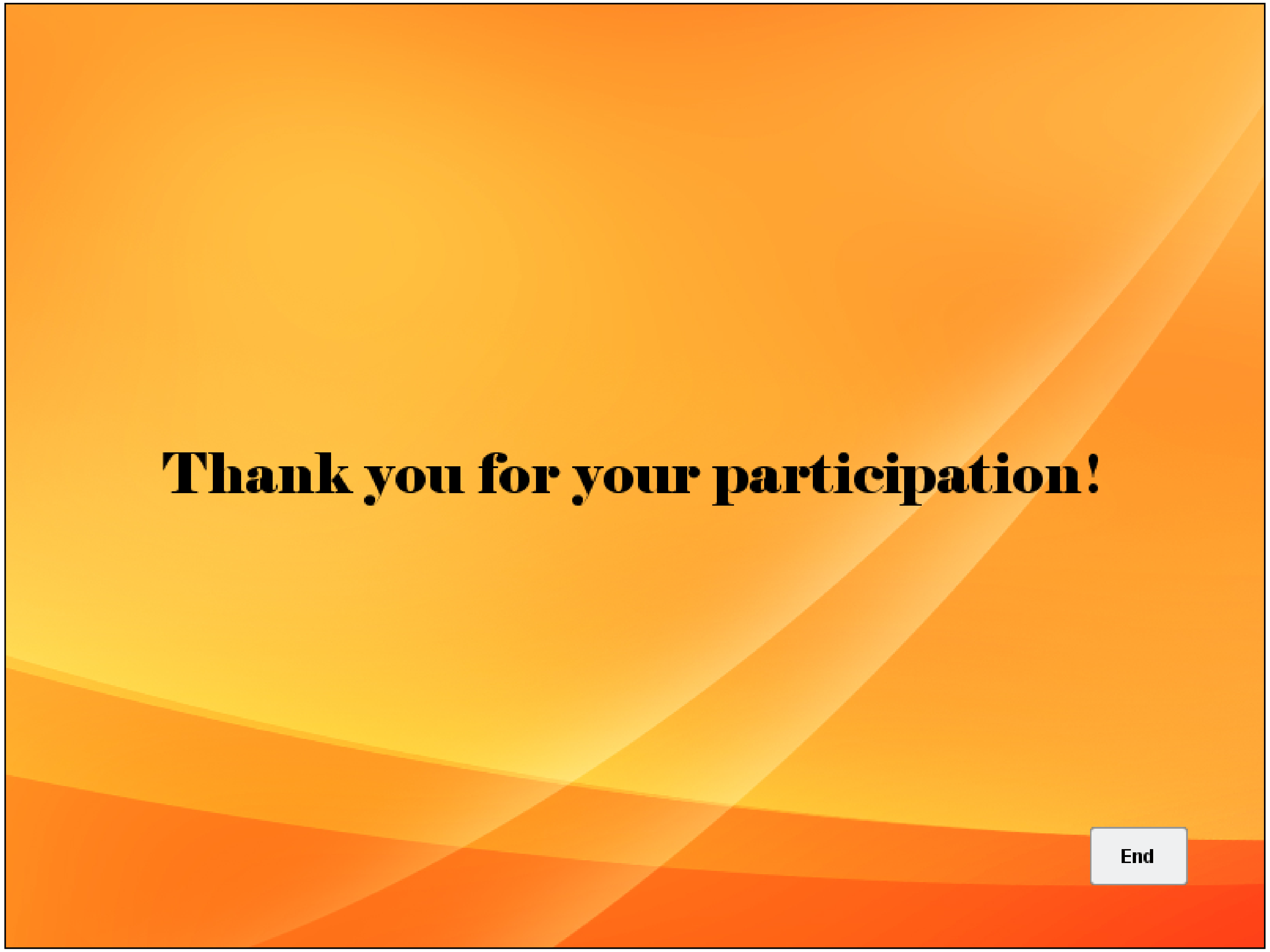

Supplement: S2 Slides — (PDF) [file pone.0203276.s006.pdf]
