# Supplementary material for: A descriptive system for the Infant health-related Quality of life Instrument (IQI): Measuring health with a mobile app
Source: PLoS One. 2018 Aug 31;13(8):e0203276. doi: 10.1371/journal.pone.0203276 (PMC6118381; doi:10.1371/journal.pone.0203276)
Supplement: S1 Table — Top: HRQoL instuments (study 1); bottom: clinical scales and index scores (study 2). (DOCX) [file pone.0203276.s007.docx]

|  | |  |  |
| --- | --- | --- | --- |
| **Search N°** | **Strategy** | | |
| 1 | Quality of life[Thesaurus] OR (quality of life or qol or hrqol or hrql or wellbeing*).ti.ab. | | |
| 2 | Questionnaire[Thesaurus] OR Self-report[Thesaurus] (questionnaire* or scale* or instrument* or survey* or measure* or parent-administ* or parent-report*).ti.ab. | | |
| 3 | Asthma[Thesaurus] or Allergy[Thesaurus] or Allergic disease[Thesaurus] or Colic[Thesaurus] or Gastrointestinal disease[Thesaurus] or Temperament[Thesaurus] or (asthma or allergy or allergic or colic or gastrointestinal intolerance or GI intolerance or sleep* or temperament).ti.ab. | | |
| 4 | Normal[EMTree] or (healthy).ti.ab. (the thesaurus term ‘Normal’ is not available in Medline) | | |
| 5 | #3 OR #4 | | |
| 6 | #1 AND #2 AND #5 AND Limits: Abstracts, Humans, English language, Last 15 years, ‘All infants (birth to 23 months)’ (for Medline) / Infant ‘to one year’ (for Embase) | | |
| .ti,ab.: search performed in the title and abstract fields *: unlimited truncation (it will find any word that begins with the term | | | |
|  |  | | |
|  |  | | |

| **Search N°** | **Strategy** |
| --- | --- |
| 1 | Health [Thesaurus] OR (performance status or clinical scale or index score or index measure or clinical scale or rating scales*).ti.ab. |
| 2 | Questionnaire[Thesaurus] OR Self-report[Thesaurus] (questionnaire* or scale* or instrument* or survey* or measure* or parent-administ* or parent-report*).ti.ab. |
| 3 | Asthma[Thesaurus] or Allergy[Thesaurus] or Allergic disease[Thesaurus] or Colic[Thesaurus] or Gastrointestinal disease[Thesaurus] or Temperament[Thesaurus] or (asthma or allergy or allergic or colic or gastrointestinal intolerance or GI intolerance or sleep* or temperament).ti.ab. |
| 4 | Normal[EMTree] or (healthy).ti.ab. (the thesaurus term ‘Normal’ is not available in Medline) |
| 5 | #3 OR #4 |
| 6 | #1 AND #2 AND #5 AND Limits: Abstracts, Humans, English language, Last 15 years, ‘All infants (birth to 23 months)’ (for Medline) / Infant ‘to one year’ (for Embase) |
| .ti,ab.: search performed in the title and abstract fields *: unlimited truncation (it will find any word that begins with the term | |
|  |  |
